# Molecular Tailoring Approach for the Estimation of Intramolecular Hydrogen Bond Energy

**DOI:** 10.3390/molecules26102928

**Published:** 2021-05-14

**Authors:** Milind M. Deshmukh, Shridhar R. Gadre

**Affiliations:** 1 Department of Chemistry, Dr. Harisingh Gour Vishwavidyalaya (A Central University), Sagar 470003, India; 2 Department of Scientific Computing, Modelling and Simulation, Savitribai Phule Pune University, Pune 411007, India

**Keywords:** hydrogen bond (HB), intramolecular hydrogen bond (IHB), molecular tailoring approach (MTA), fragmentation methods, bond energy estimation, noncovalent interactions

## Abstract

Hydrogen bonds (HBs) play a crucial role in many physicochemical and biological processes. Theoretical methods can reliably estimate the intermolecular HB energies. However, the methods for the quantification of intramolecular HB (IHB) energy available in the literature are mostly empirical or indirect and limited only to evaluating the energy of a single HB. During the past decade, the authors have developed a direct procedure for the IHB energy estimation based on the molecular tailoring approach (MTA), a fragmentation method. This MTA-based method can yield a reliable estimate of individual IHB energy in a system containing multiple H-bonds. After explaining and illustrating the methodology of MTA, we present its use for the IHB energy estimation in molecules and clusters. We also discuss the use of this method by other researchers as a standard, state-of-the-art method for estimating IHB energy as well as those of other noncovalent interactions.

## 1. Introduction

The hydrogen bond (HB) is a dominant noncovalent interaction found in chemical and biological systems [1,2,3,4]. The term “hydrogen bond” seems to have emerged around 1930, from the works of Pauling [1] and Huggins [5,6]. However, the mention of weak, yet specific interactions involving the hydrogen atom is much older. The dimeric association of molecules with hydroxyl groups was suggested by Nernst in 1892 [7]. The term “Nebenvalenz” by Werner [8] and “weak union” by Moore and Winmill [9] are other early stipulations of this noncovalent interaction. In 1920, Latimer and Rodebush [10] suggested that the hydrogen nucleus in an aqueous solution of amines is held jointly by two octets, constituting a weak bond. Barnes, while studying the structure of ice [11], suggested that the hydrogen atoms were midway between the two oxygen atoms, though he did not explicitly mention the hydrogen bond. Huggins [12] claimed that he was the first to propose the term “H-bond” in 1919. His later usage of the term “hydrogen-bridge” may have led to the German word “Wasserstoffbrücke.” The concept of HB gained popularity after Pauling published his classic book, *The Nature of the Chemical Bond* in 1939 [1]. Pimentel and McClellan [13] suggested that an HB exists when (i) there is evidence of a bond and (ii) there is evidence that this bond involves a hydrogen atom already bonded to another atom. The recent definition of HB by IUPAC [14] is similar in spirit to that in Ref. [13]. The former [14] states that “the hydrogen bond is an attractive interaction between a hydrogen atom from a molecule or a molecular fragment X–H in which X is more electronegative than H, and an atom or a group of atoms in the same or a different molecule, in which there is evidence of bond formation.”

An HB may be generally represented as X–H···Y, where X–H is the proton donor and Y is a proton acceptor. The X–H···Y interactions such as O–H···O, N–H···O, N–H···N, S–H···O, etc., in neutral molecular systems, exhibit interaction energies lying between ~1 to 20 kcal/mol. Typical H···Y distances suggested in the literature fall in the range of ~1.2–3.0 Å and X–H···Y angles lie between 100 and 180° [2,3,4]. The HB’s in liquid water are central to water’s life-providing properties [1,15]. It is stipulated in the literature [16] that if HBs in water were 7% stronger or 29% weaker, water would not be a liquid at room temperature. HBs provide a significant driving force for the native structures and functions of biomolecules [17,18]. Hence, it is of great importance to reliably estimate these X−H···Y HB strengths for shedding light on several physicochemical phenomena and life processes.

The theoretical estimation of intermolecular X–H···Y HB strength in a complex A···B is routinely performed using a supermolecular approach, in which the HB energy (E_HB_) is estimated as E_HB_ = E_A···B_ − (E_A_ + E_B_). Several methods for estimating the intermolecular interaction energies are reported in the literature (see, e.g., Refs. [19,20,21,22]). On the other hand, quantifying an intramolecular hydrogen bond (IHB) strength is not as straightforward as the intermolecular one. The main difficulty lies in isolating the X–H···Y interaction present within a molecule than in a dimer or a complex. Many studies for gauging the strength of the IHB in the literature are based on spectroscopic- [23,24,25,26] and electron density topological approaches [27,28,29,30]. Nevertheless, some empirical, semiempirical, and ab initio procedures [31,32,33,34,35,36,37,38,39] have also been reported in the literature for estimating the IHB energy. These have been nicely summarized by Jablonski [40] in his article in this Special Issue, and we shall discuss only the aspects of these methods (e.g., their merits and demerits) that are not explicitly covered in Ref. [40].

One of the early approaches is the conformational analysis (CA), in which two different conformers of the reference molecule are considered. These conformers are chosen such that the HB is kept intact in one of the conformers and is broken in another. The energy difference between these two conformers is then taken as the measure of IHB energy [32,40,41]. A significant disadvantage of this method is that the estimated IHB energy is erroneous due to the incorporation of attractive (*syn-anti*) or repulsive (*anti-anti*) additional interaction in one of the conformers [42]. In another similar procedure, viz., the *ortho-para* method [43], the IHB energy of the X-H···Y bond formed by two substituents, which are *ortho* to each other, is taken as the energy difference between the *ortho* and *para* forms of the reference molecule. However, this method applies only to aromatic systems in which an HB is present in two substituents, which are *ortho* to each other. The main drawback of this method is that it assumes that the electronic effects caused by the substituent at different positions are similar between the *ortho*- and *para*-conformers [44,45,46].

Yet, another indirect approach is the isodesmic/homodesmic reactions. In the former, the IHB making/breaking reaction is written so that the number and type of bonds on either side of the reaction are equal, except the HB, which is retained in one of the reactants [33,47,48]. A further assumption is that the atomic hybridization is conserved on both sides of the reaction. In that case, the method is called homodesmic reaction, which is supposed to give more reliable energy estimates than the isodesmic reaction [49,50,51]. The main drawback of the isodesmic/homodesmic reaction approach is that it does not give HB energy but includes strain energy due to the formation of a ring structure [52]. Another major disadvantage of these indirect methods is that they are applicable only to the evaluation of energy of a single HB present in the system and cannot be employed in a system containing multiple HBs.

Another popular but indirect method is based on the quantum theory of atoms in molecules (QTAIM) [53]. In this method, the presence of a (3, −1) bond critical point (BCP) of the molecular electron density (MED) between H···Y, is considered as the signature of an HB. The large/small value of the MED at the BCP is seen to correlate with strong/weak X–H···Y interaction [54,55,56,57,58]. Espinosa et al. [59] proposed an empirical relation, *E*_HB_ = 0.5 V(**r**_cp_), where V(**r**_cp_) is the potential energy density at BCP. Interacting quantum atoms (IQA) [60] framework, leading to QTAIM-compatible energy partition, is another indirect approach wherein the HB energy is calculated as the sum of the classical Coulombic interaction between groups involved in the HB and the exchange-correlation energy. It has been pointed out that there is no check on the reliability of HB energy provided by both of these methods [61,62]. Further, these empirical equations are applicable only when a (3, −1) BCP is present. For instance, a (3, −1) BCP at O–H···O bond is conspicuous by its absence in all the polyols having an O–H···O interactions between the vicinal -OH groups. However, the weak O–H···O HB was confirmed in ethylene glycol-based on the vapor phase OH-stretching overtone spectroscopy [63,64,65]. Some other variants and indirect empirical equations proposed in the literature for estimating the strength of IHBs are summarized in Ref. [40]. Hence, we skip the discussion of these methods.

A brief review of the above literature suggests that these empirical and indirect methods for estimating IHB energy are generally limited to singly H-bonded systems. These cannot be readily extended to a system containing multiple HBs and hence also to the estimation of HB cooperativity due to an interconnected network of HBs. Most importantly, there is no check on the reliability of the estimated HB energies. Thus, it was felt necessary to come up with a direct theoretical method for a reliable estimation of IHB energy. Deshmukh and Gadre [66,67] proposed such a procedure, based on the in-house developed molecular tailoring approach (MTA) for the ab initio treatment of large molecular systems [68,69,70,71,72,73,74,75,76,77,78,79,80,81,82]. The MTA currently enables the calculation of one-electron properties, geometry optimization, and the calculation of vibrational infrared and Raman spectra of large molecules/clusters using DFT or correlated methods. Before discussing the application of MTA for the IHB energy estimation, we explain the working principle of MTA, with an illustrative example, in Section 2.

## 2. Molecular Tailoring Approach

The molecular tailoring approach (MTA) is a fragmentation-based technique developed by Gadre et al. [68,69,70,71,72,73,74,75,76,77,78,79,80,81,82]. Within MTA, a spatially extended molecular system under consideration is partitioned into a set of overlapping fragments (called the “main fragments”) on which ab initio calculations for one-electron property or the energy are carried out. The fragmentation may be carried out automatically or manually. The quality of the fragmentation scheme is gauged by a parameter called R-Goodness (Rg), which may be estimated as follows: Put a sphere of radius R centered on a reference atom i so that all the atoms of the parent system lying within this sphere belong to at least one of the main fragments. The maximum value of R obeying this condition is called the Rg value of atom i in the given fragmentation scheme. The minimum of such atomic Rg values is called the Rg value of the scheme. In general, the larger the Rg value of a fragmentation scheme, the better is the chemical environment of each atom mimicked [69]. After choosing an appropriate fragmentation scheme, molecular energy E, of the spatially extensive parent system is estimated approximately by patching those of the individual fragment energies [69] using Equation (1).

(1) $$E=\sum_{i}E^{F_{i}}-\sum_{i<j}E^{F_{i}\cap F_{j}}+\cdot \cdot \cdot +{\left(-1\right)}^{k}\sum_{i<j<\cdot \cdot \cdot <n}E^{F_{i}\cap F_{j}\cap \cdot \cdot \cdot \;\cap F_{n}}$$

where the energy E of the parent molecule is estimated as the sum of energies of primary fragments {$F_{i}\}\;$ minus the sum of energies of binary overlap fragments {$F_{i}\cap F_{j}\}$ plus the sum of energies of ternary overlap fragments, etc. Here, k stands for the degree of overlap., e.g., for binary overlap, k = 1, for ternary overlap, k = 2, etc. Equation (1) is generalized for estimating an electronic property of the molecule, such as the energy gradients, the Hessian matrix elements, etc.

**Scheme 1:**
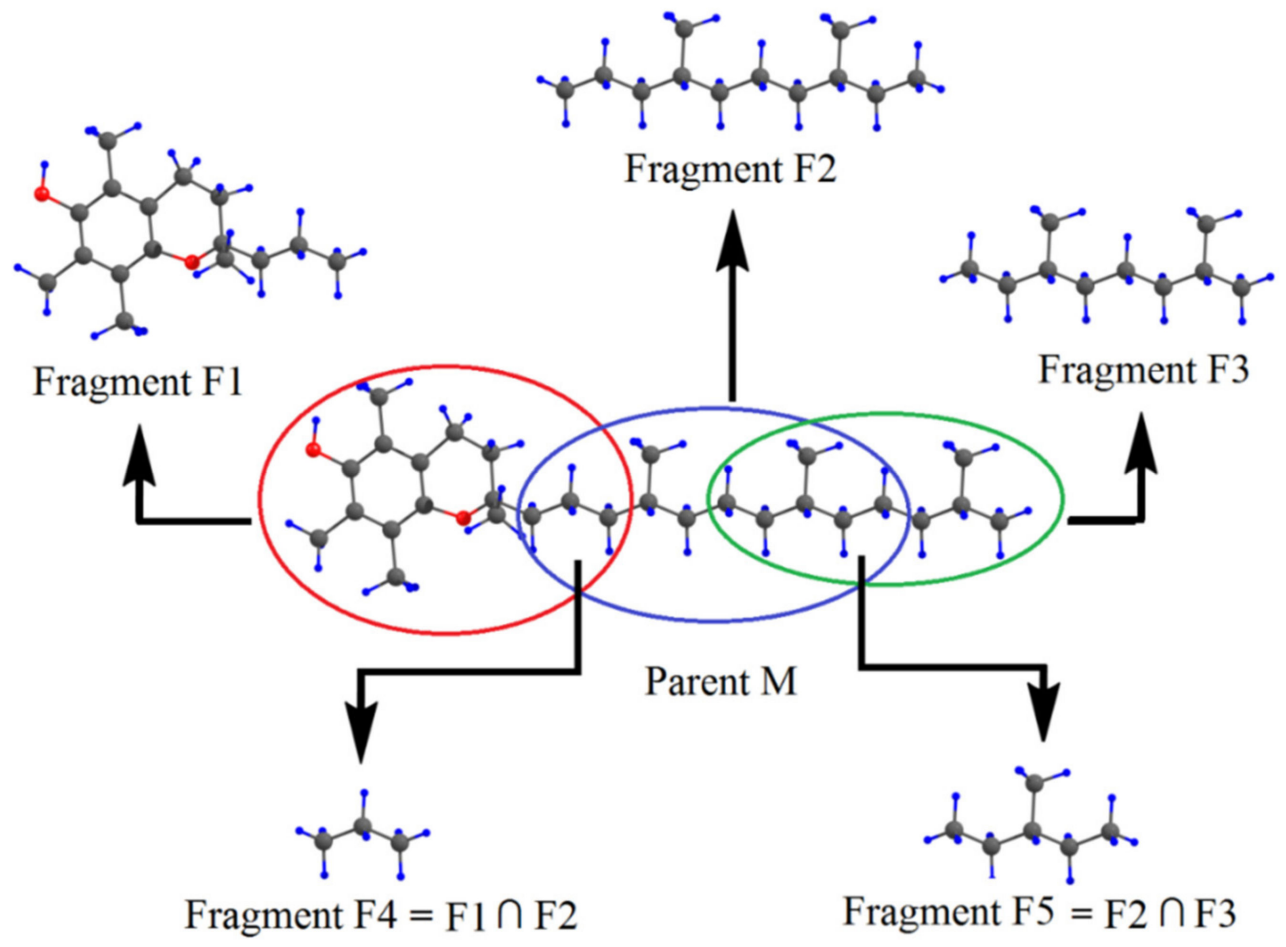
Illustration of the molecular tailoring approach (MTA)-based fragmentation procedure for α-tocopherol, shown as the parent molecule, M. See text for details.

We now illustrate the fragmentation procedure with a test example, viz., the α-tocopherol molecule (shown as M) in Scheme 1, fragmented into three primary fragments F_1_, F_2_, and F_3_ (shown by appropriate circles). The fragments F_4_ and F_5_ are the binary overlaps of fragments F_1_, F_2_, and F_2_, F_3_, respectively. Here, the term binary overlap means the common structural part of two primary fragments. In fragmentation Scheme 1, the ternary fragment (overlap of three fragments F_1_, F_2,_ and F_3_) is absent. The valencies of the cut regions are satisfied by placing the H-atoms at the appropriate C–H distance of 1 Å along the cut C–C bond. The calculations for the single point energy (or the property) of these fragments F_1_ to F_5_ are performed. For this test case, we report the MTA energy calculation of M at HF/6-31+G(d,p) level theory using Equation (1). It should be noted that the HF method employed here is only for illustrative purposes. MTA method works at any correlated level of theory. All the calculations are performed with the Gaussian 16 package [83]. 

In the present case, the energy (E_MTA_) of the parent molecule (M) is obtained by Equation (2) as

E_MTA_ = {E_F1_ + E_F2_ + E_F3_} − {E_F4_ + E_F5_} (2)

In the present case of α-tocopherol this energy is calculated utilizing the energies of the fragments as E_MTA =_ {(−769.56869) + (−469.57421) + (−391.51579)} − {(−118.26152) + (−235.36444)} = −1277.03273 hartrees (a.u.). The actual energy (E_FC_) at the HF/6-31+G(d,p) level of theory is E_FC_ = −1277.03288 a.u. Thus, the error (E_Error_) in the estimation of molecular energy is given E_Error_ = E_FC_ − E_M_ = −0.00016 a.u. This error can be further reduced using the so-called grafting procedure embedded in the current version of MTA [77,78].

Gadre et al. first proposed the MTA methodology for estimating the electrostatic properties of large, closed-shell molecules [68]. However, in the last 24 years, the scope of MTA was extended for the estimation of molecular energy [69], geometry optimization [70,71], the estimation of the Hessian matrix [72], the computation of vibrational infrared [73] and Raman spectra [74], and binding energies of large molecular clusters and complexes [75,76,77,78,79,80,81,82]. Since the fragment computations are independent of each other, MTA has the advantage that the energy computation of the parent molecule is intrinsically parallel. Further, MTA can currently work with Gaussian, GAMESS, and NWChem at the back-end, thereby becoming a powerful tool for ab initio treatment of large molecules and clusters, when used in conjunction with a high level of theory, such as MP2 or CCSD(T) employing a large basis set. One important application of MTA is in estimating the IHB energy [52,66,67,84,85,86,87,88,89,90,91,92]. This will be discussed in the next section.

## 3. Intramolecular Hydrogen Bond Energy Estimation by Molecular Tailoring Approach

With the above brief introduction to MTA, we now discuss its application for estimating the IHB energy. As discussed in the introduction section, intermolecular X–H···Y HB energy in a complex A···B is estimated as E_HB_ = E_A···B_ − (E_A_ + E_B_). This estimation is possible because the energies of the two monomers A and B can be separately calculated. In the case of intramolecular X–H···Y interaction, such a separation is, in general, difficult. However, the MTA procedure allows the generation of fragments so that the atoms/functional groups involved in the HB formation are parts of two different fragments.

**Scheme 2:**
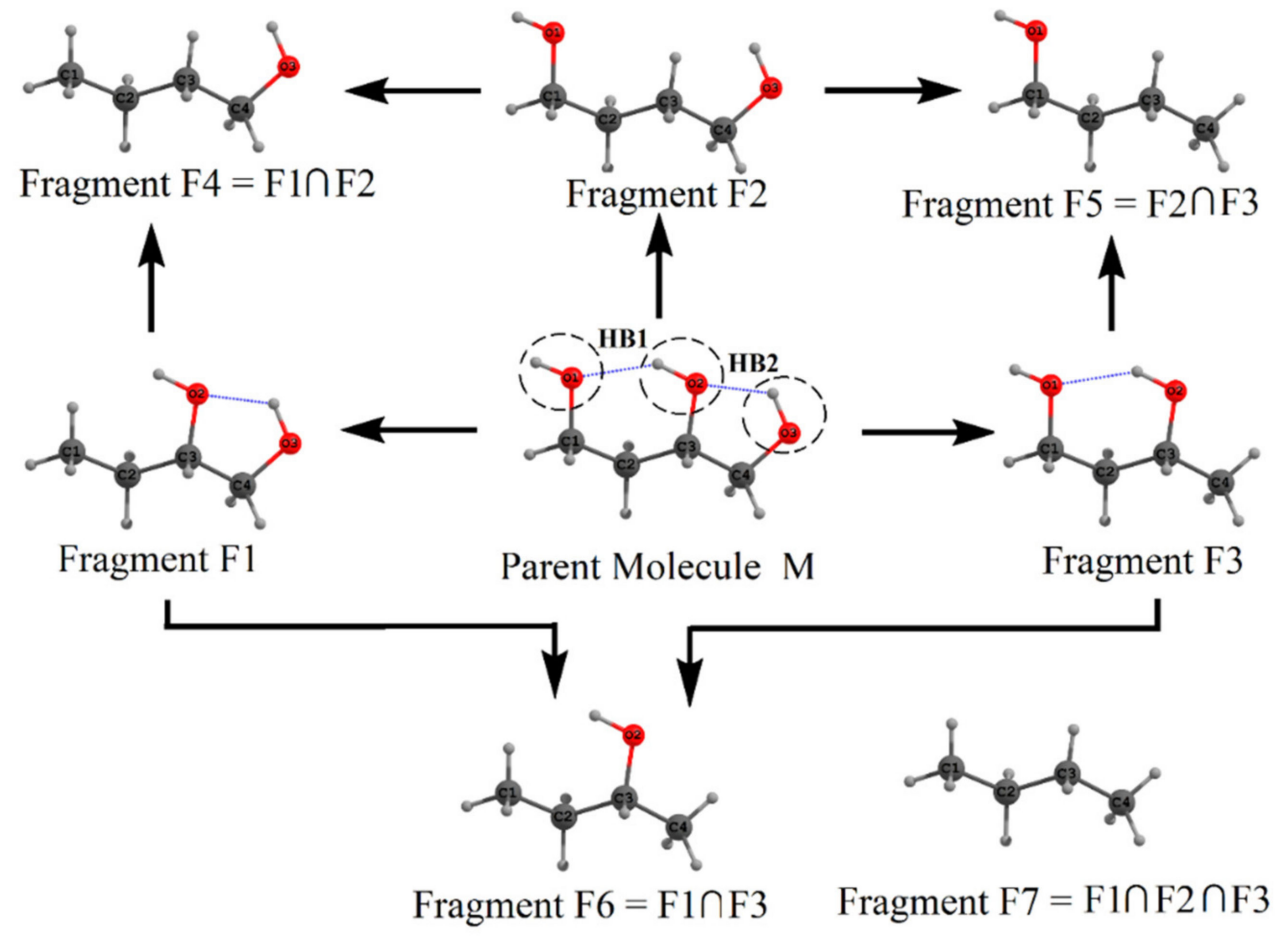
Fragmentation procedure for estimating the energies of the H-bonds, **HB1**, and **HB2** in 1,2,4-butanetriol (Parent M) molecule. See text for details.

The fragmentation procedure is illustrated in Scheme 2 for the test molecule of 1,2,4-butanetriol. In Scheme 2, the parent test molecule, denoted as M, is shown at the center. The geometry of 1,2,4-butanetriol was optimized at the MP2/6-31+G(d,p) (default option: frozen core) level of theory using the Gaussian package [83]. The energy of the optimized structure is −383.01926 a.u. at MP2/6-31+G(d,p) level. The three oxygen atoms are shown as O1, O2, and O3 (see Scheme 2), with the two HB’s, viz., **HB1** (O2-H···O1) and **HB2** (O3-H···O2) whose energy is to be estimated. For this purpose, the parent molecule is cut into three primary fragments F1, F2, and F3, obtained by replacing −O1H, −O2H, and −O3H groups, respectively, with an H atom each. Dotted circles show these cut regions on the original molecule. The H-atoms are added along the respective C–O bonds (which are cut to form these primary fragments) so that the C–H distance is 1 Å. Hydrogen is the simplest monovalent atom that can be used for satisfying the valencies of cut regions. It is emphasized here that H-atoms placed at slightly different distances (say at 0.9 or 1.1 Å) from the C-atom do not change the results appreciably. This is because of the cancellation of errors in estimating the molecular energy using these fragments. Fragments F4, F5, and F6 are obtained by taking the binary intersection of these primary fragments, i.e., (F1∩F2), (F2∩F3), and (F1∩F3), respectively. Here, intersection means the common structural parts between two primary fragments apart from added H-atoms. For instance, in fragment F4, C1(H_2_)–C2(H_2_)–C3(H_2_)–C4(H_2_)O3(H) is the common structural part that is also present in fragments F1 and F2. Similarly, fragment F7 is the common intersection of three primary fragments F1, F2, and F3, i.e., (F1∩F2∩F3). A single point energy evaluation, at MP2/6-31+G(d,p) level of theory, is carried out on all seven fragments obtained by the above fragmentation procedure. The fragment geometries are not optimized to avoid the conformational changes in them so that they lead to reliable estimates of IHB energies. It is necessary first to provide a check on MTA application to the parent molecule, M. As discussed above, the actual energy of the original molecule (M) is *E*_M_ = −383.01926 a.u. at the MP2 level of theory. Using the MP2 single point energies of these fragments, the estimated molecular energy of M is: E_M_ = {E_F1_ + E_F2_ + E_F3_} − {E_F4_ + E_F5_ + E_F6_} + E_F7_ = {−307.97304 + (−307.9642) + (−307.97765)} − {−232.92410 + (−232.92514) + (−232.93273)} + (−157.88552) = −383.01844 a.u. The error, ΔE = |MTA energy - actual energy| in molecular energy indeed turns out to be very small, viz., 0.00082 a.u. This excellent agreement between the MTA-estimated and actual energy suggests that the present fragmentation scheme is reliable for evaluating HB energies.

Now we estimate the energy of two hydrogen bonds **HB1** and **HB2,** in the parent 1,2,4-butanetriol. Recall that the hydroxyl groups involved in the formation of hydrogen bond **HB1** are O1–H and O2–H. These hydroxyl groups were replaced in fragments F1 and F2, respectively, by H-atoms. Putting the geometry of fragment F1 over F2, we regenerate the parent molecule except following two things: (i) the O–H···O H-bond, i.e., the **HB1** interaction between O1–H and O2–H present in the parent molecule is missed out and (ii) there is double counting of common structural part between F1 and F2 (viz., the secondary fragment, F4). Upon addition of single-point energies of fragments F1 and F2, followed by subtraction of the energy of fragment F4 would give the energy of the parent molecule except that the energy of the HB, viz., **HB1** is missed out. If the energy of the parent 1,2,4-butanetriol E_M_ is subtracted from (E_F1_ + E_F2_ – E_F4_), the HB energy E**_HB1_** is obtained as E**_HB1_** = (E_F1_ + E_F2_ − E_F4_) − E_M_ = 3.84 kcal/mol. In a similar fashion, E**_HB2_** is obtained as E**_HB2_** = (E_F2_ + E_F3_ − E_F5_) − E_M_ = 1.60 kcal/mol. It should be noted here that these estimated HB energies are in the gas phase. However, the MTA-based method in principle can provide HB energies in the solvent phase, wherein the energies of the fragments in solvent (using continuum solvation model) could be employed.

We note that the two HBs, **HB1** and **HB2**, are interconnected, forming an H-bond network. Such networking of H-bonds leads to a phenomenon called cooperativity [67]. In general, it is anticipated that the strengths of **HB1** and **HB2** are enhanced because of this networking effect. To estimate the contribution of cooperativity toward each of these two H-bonds, we reestimated the HB energy of these two HBs by isolating them from each other. The difference between the HB energy estimated earlier (in the presence of network) and the one when they are isolated (in the absence of a network) is the cooperativity contribution toward this HB. For example, consider fragment F3 in which only **HB1** is present and fragment F1 in which **HB2** is present. To estimate the energy of **HB1** in the absence of the networking effect of **HB2**, we consider fragment F3 as our parent molecule. In the present case, fragments F5 and F6 are the two primary fragments that, when placed over each other, would give us the parent fragment F3 except **HB1**, and fragment F7 is the binary overlap of F5 and F6. Therefore, utilizing these fragments’ energies, the energy of **HB1** is obtained as E_HB1_ = (E_F5_ + E_F6_ − E_F7_) − E_F3_ = 3.32 kcal/mol. Similarly, the energy of **HB2** in the absence of the networking effect of **HB1** is obtained as E_HB2_ = (E_F4_ + E_F6_ − E_F7_) − E_F1_ = 1.09 kcal/mol. These reestimated HB energies are indeed smaller than those estimated in the presence of the networking effect. The difference in the energy is cooperativity contribution. The cooperativity contribution to **HB1** is $E_{\mathrm{HB}1}^{\mathrm{coop}}$= 3.84 − 3.32 = 0.52 kcal/mol and that for **HB2** is $E_{\mathrm{HB}2}^{\mathrm{coop}}$= 1.60 − 1.09 = 0.51 kcal/mol. In the present test case, the estimated cooperativity contributions are not large because only two HBs are present. The later sections will show that cooperativity values in some molecules can indeed be as large as a typical HB energy.

The HB energies obtained by applying the above procedure to some alkanetriol molecules are shown in Table 1 [66]. The estimated HB energies fall in a range between 1.50 and 4.97 kcal/mol (see Table 1). This is the expected energy range from chemical intuition. Further, these HB energies are in a qualitative agreement with those expected from the corresponding HB distances. For instance, the strongest HB in 1,2,5-pentanetriol has an energy of 4.97 kcal/mol, with the corresponding HB distance being the shortest (1.80 Å) among all the alkanetriols reported in Table 1. One of the noteworthy results in Table 1 is that the error in estimating molecular energies of all the alkanetriols is quite small, viz., between 0.40 to 0.65 kcal/mol. By considering this accuracy, we estimate the maximum error associated with our calculated HB energies to be 0.3 kcal/mol. The present method is thus capable of calculating accurately the IHB energies and cooperativity values of multiply H-bonded systems. This is a significant advantage of the current method over the other indirect approaches reported in the literature.

## 4. Critical Comparison of MTA with Other Methods

We now compare the estimated HB strengths in these molecules with those qualitatively estimated by other indirect measures. These measures include the O–H stretching frequency, molecular electron density (MED) value at (3, −1) BCP, and the HB energy estimated using the isodesmic reaction approach (IDRA) and that by using Espinosa’s equation. Both the MED value at (3, −1) BCP and the shift in the stretching frequency of the O–H involved in the HB show a good qualitative correlation with the estimated HB energies (see Table 1). For instance, the strongest HB found in 1,2,5-pentanetriol (4.97 kcal/mol) corresponds to the highest MED value (0.0334 au) at the (3, −1) BCP. The stretching frequency of the O–H bond involved in the HB is 3669 cm^−1^, showing a redshift. The weakest HB is seen in 2,3,4-pentanetriol (1.50 kcal/mol), with the (3, −1) BCP being absent and a large O–H stretch frequency of 3820 cm^−1^. In general, the calculated HB energies show a good qualitative rank–order relationship with the corresponding O–H stretching frequencies and the MED value at the respective (3, −1) BCP.

**Scheme 3:**
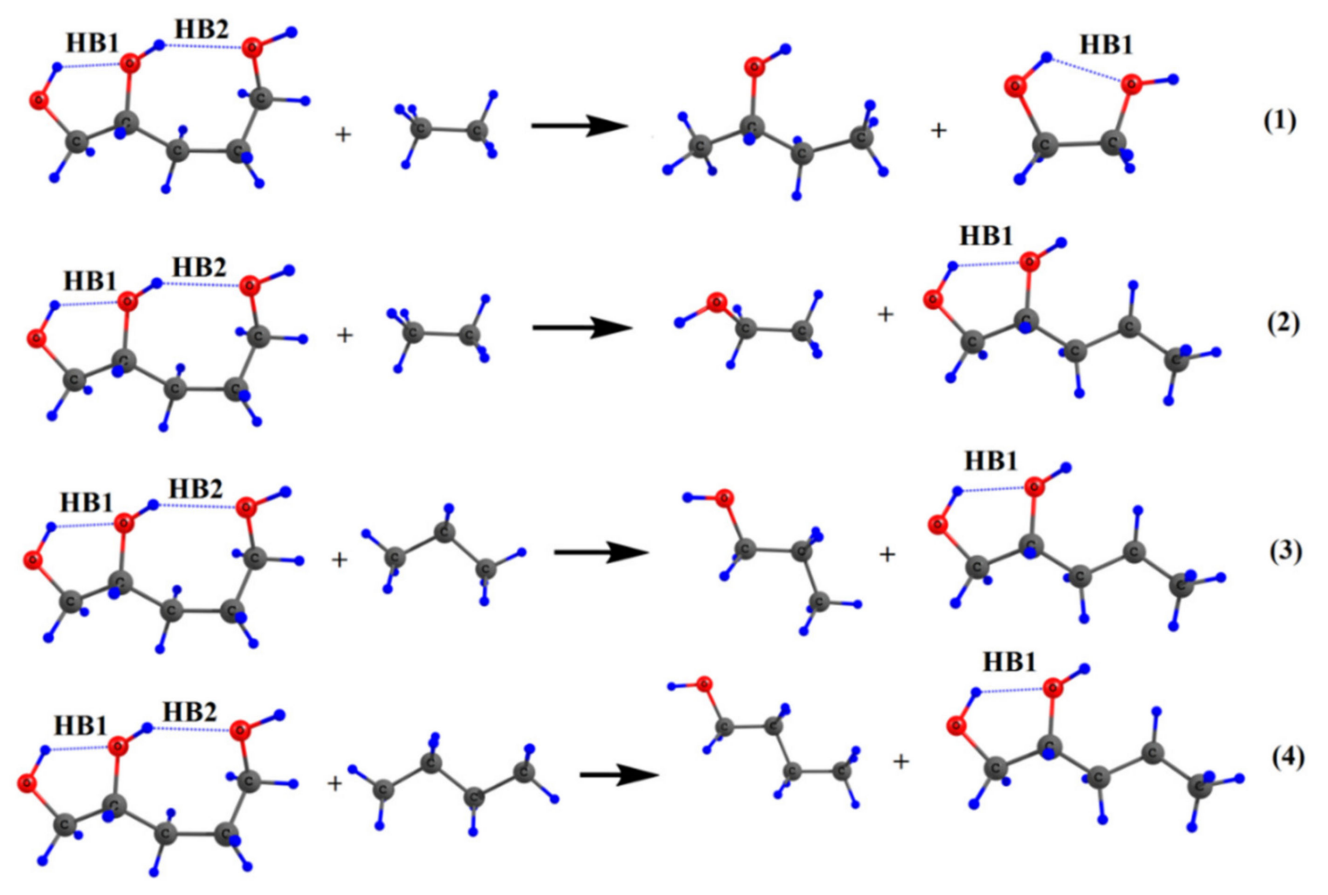
Some possible isodesmic reactions for the estimation of H-bond energy, E**_HB2_** in 1,2,5-Pentanetriol. See text and Ref. [52] for details.

We now critically compare our MTA-based results with those obtained by yet another indirect method, viz., the isodesmic reaction approach (IDRA) [33,48]. In IRDA, the IHB-making/breaking reaction is written such that, except for the HB under consideration, the number and type of other bonds are conserved on both sides of the reaction. Within IDRA, all the reactant and product geometries are optimized at the appropriate level of theory. The energy change for such a reaction is taken as the HB energy. The main disadvantage of IDRA is that there is no unique way of writing an isodesmic reaction for estimating the HB energy. For example, in Scheme 3, four possible reactions are shown for evaluating the HB energy E**_HB2_** in 1,2,5-pentanetriol, which retain **HB1** on both sides of these isodesmic reactions. See Ref. [52] for details of several other isodesmic reactions for estimating the HB energies of **HB1** and **HB2**.

**Figure 1:**
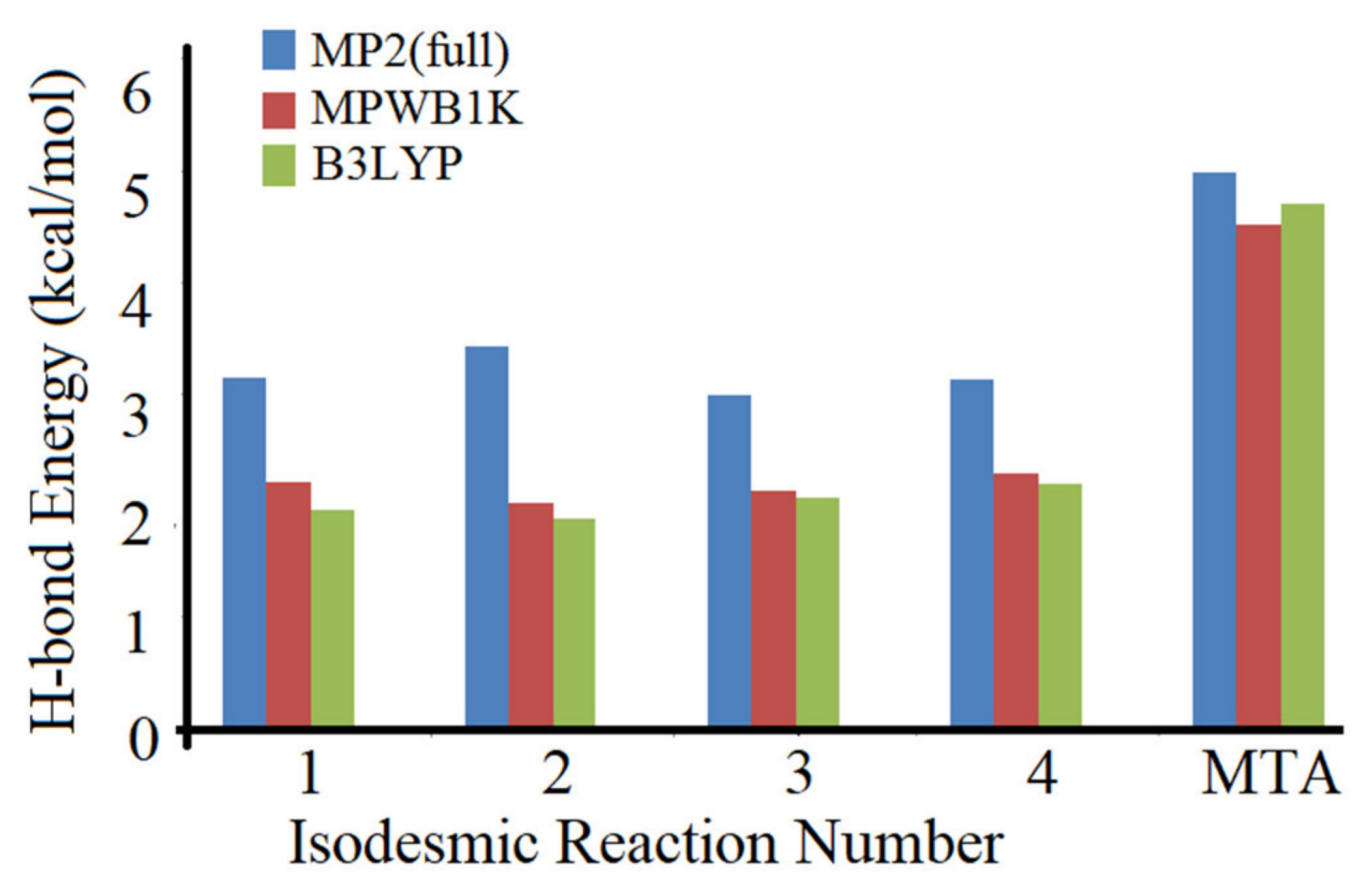
The H-bond energy, E**_HB2_**, in 1,2,5-pentanetriol calculated at different levels of theory using isodesmic reactions (see Scheme 3) and also by molecular tailoring approach (MTA). See text for details.

Figure 1 shows a histogram of the estimated HB energy E**_HB2_** by these four reactions and also by the MTA-based method. The HB energies estimated by the isodesmic reactions vary significantly across the levels of theory and from each other. These estimated energy values are much smaller (30 to 40%) than the respective MTA-based ones. The reasons for these smaller HB energy values by IDRA can be understood as follows: In IDRA reactions presented in Scheme 3, on the reactant side, the **HB2** bond formation between the -OH groups at C2 and C5 positions leads to a seven-membered ring-like structure involving one O-H···O, two C-O, and three C-C bonds. This ring formation has the ring strain effect in the parent 1,2,5-pentanetriol molecule. Since the reactant and product geometries are optimized, this ring strain effect is not preserved on the product side of these reactions. Therefore, the molecules on the product side are more relaxed, losing most of their ring strain due to the loss of the **HB2** bond. It may be noted here that the ring strain may be small or canceled out when one estimates the energy of **HB1** using IDRA. This is because the formation of **HB1** leads to the formation of a five-membered ring-like structure involving one O-H···O, two C-O, and only one C-C bonds. This ring (five-membered) backbone is expected to be maintained (to some extent if not fully) on both reactant and product sides as it involves only one C-C bond. For more details about **HB1** energy by IDRA, see Ref. [52].

Thus, the finer bonding effects are not mimicked evenly on both sides of the reaction, resulting in poor estimates of HB energy by IDRA. On the contrary, in the MTA method, the geometry of the fragments is not optimized, and the fragment backbone structure is preserved, leading to accurate estimates of the HB energies. For instance, the evaluation of HB energy E**_HB1_** as (E_F1_ + E_F2_ − E_F4_) − E_M_ in 1,2,4-butanetiols involves the energies of F1, F2, and F4 (see Scheme 2). Thus, the MTA-based method leads to unique isodesmic/homodesmic reaction, viz., M + F4 → F1 + F2. As seen in Scheme 2, the carbon backbone structure is retained on either side of this unique reaction. In other words, as advocated in the literature [49,50,51,52], IDRA does not yield the true HB energies. Another drawback of the IDRA is for estimating the HB strengths in multiply hydrogen-bonded systems. Here, one has to write different reactions for different HBs. In summary, the MTA-based method for the estimation of IHB energy is accurate and can be applied to the evaluation of multiple IHB energies in a given molecule. In the following sections, we present and discuss the results of IHB energies obtained by applying the MTA-based method to a variety of systems.

We now present a comparison of the HB energy estimated by the MTA-based method with those by Espinosa’s empirical relation [59]. For this purpose, we consider two molecular systems having O–H···O=C HB, viz., 2′-Hydroxyacetophenone, and methyl 2-hydroxybenzoate. The geometries and HB energies by Espinosa’s method in these two molecules were taken from the Ref. [93]. The reported HB energies at B3LYP/6-311++G(d,p) level, by Espinosa’s method, in 2′-Hydroxyacetophenone and methyl 2-hydroxybenzoate are 15.3 and 11.4 kcal/mol, respectively. The HB energies for these molecules were calculated by us at the given geometries, at the same level of theory, by MTA-based method are 9.7 and 7.8 kcal/mol, respectively. As can be observed, the HB energies estimated by Espinosa’s method are significantly overestimated, compared to the HB energies by the MTA method. These results are in agreement with an earlier report that the use of Espinosa’s method significantly overestimated the energy of HBs [62,93]. Although our MTA-based method requires extensive calculations and more computational time, reliable direct estimates of the HB energies are thereby obtained. Further, the internal benchmarking of the total energy is possible for the MTA-based method. Such benchmarks are not available in Espinosa’s method.

## 5. Application to Large Molecules and Clusters with Multiple Hydrogen Bonds

We now discuss the application of the MTA-based method for IHB energy estimation in several large systems. One such interesting system is a class of carbohydrates, viz., sugar molecules. These molecules play an important role in biological processes, which are mainly governed by weak interactions such as hydrogen bonding, hydrophobic effects, etc. Hence, understanding the interactions such as H-bonding is of utmost importance. Further, the O–H groups in carbohydrates form a network of interconnected O–H ···O HBs. It is suggested that the strengths of these individual O–H···O HBs are enhanced due to such networking of HBs. 

**Figure 2:**
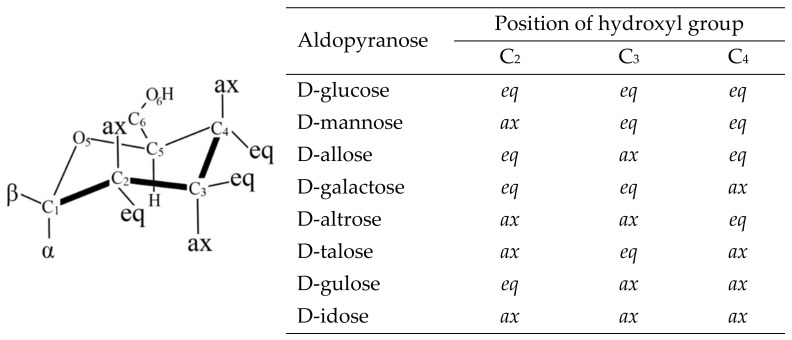
General structure of the aldopyranose sugar. In Table, *ax* represents axial, and *eq* represents equatorial orientations of the hydroxyl group at carbons C_2_-C_4_. Figure partially reproduced from Ref. [67] with the permission from American Chemical Society (ACS). *Copyright (2008) The American Chemical Society*.

In our earlier work [67], the IHB energies and the contribution to cooperativity were investigated in eight aldopyranose monosaccharides, which vary in the position of hydroxyl groups [axial (*ax*) or equatorial (*eq*)], as shown in Figure 2. The estimated HB energies are in the range of 1.2 to 4.1 kcal/mol at the MP2(full)/6-311++G(2d,2p) level of theory. It is found that the OH···O *eq-eq* interaction energies are between 1.8 and 2.5 kcal/mol, with axial-equatorial ones being stronger (2.0 to 3.5 kcal/mol). The strongest bonds involve nonvicinal *ax-ax* O–H groups (3.0 to 4.1 kcal/mol). The cooperativity contribution to the HBs is seen to fall between 0.1 and 0.6 kcal/mol for *eq-eq* HBs and is seen to be higher (0.5 to 1.1 kcal/mol) for *ax*–*ax* HBs. This work [67] was one of the first attempts for estimating the IHB energies and the respective cooperativity contributions in sugar molecules.

A similar class of compounds having no ring oxygen atom is hexahydroxy–cyclohexane, called inositol. Inositol derivatives function as intracellular signal transduction molecules, playing an important role in biological processes. It is hence important to understand the structure and stability of various inositol conformers [84]. On applying the MTA-based method, the estimated HB energies in the presence of a cooperative HB network are seen to be in the range of 2.2 to 3.8 kcal/mol at the MP2/6-311+G(d,p) level of theory. The sum of all the HB energies in these conformers of inositol falls between 7.2 to 18.1 kcal/mol. The total cooperativity contribution in these conformers is rather large, between 2 to 5 kcal/mol. It increases on going from isomers with more *eq* O-H groups to those with more *ax* ones. Importantly, the highest stability of the *scyllo* isomer in the solvent was attributed [84] to weaker intramolecular OH···O HBs between *eq* hydroxyl groups. It is suggested that these weak OH···O HBs in *scyllo* isomer may facilitate favorable intermolecular interactions with solvent molecules. In contrast, the inositol isomers with *ax* O-H groups are involved in the formation of relatively strong HBs. Therefore, they are less stable due to large steric factors in the gas phase and unfavorable intermolecular interaction in the solvent phase.

The MTA-based method was also employed for understanding the conformational stability of fructose [85]. The experimental rotational spectroscopic studies suggest that the molecules of fructose and ribose preferentially adopt the β-pyranose structure in the gas phase. It was noted that [85] the relative stability of different conformers of fructose in the gas phase could be explained in terms of three collective effects: (i) the sum of HB energies in a given conformer, (ii) the strain energy of a bare fructose ring, and (iii) the sum of anomeric stabilization (*endo* + *exo*) energies. It was concluded [85] that the small ring strain, sufficiently large sum of the IHB energies, and the higher stabilization due to anomeric interactions in β-fructo-pyranose makes it a conformationally locked predominant structure in the gas phase.

**Figure 3:**
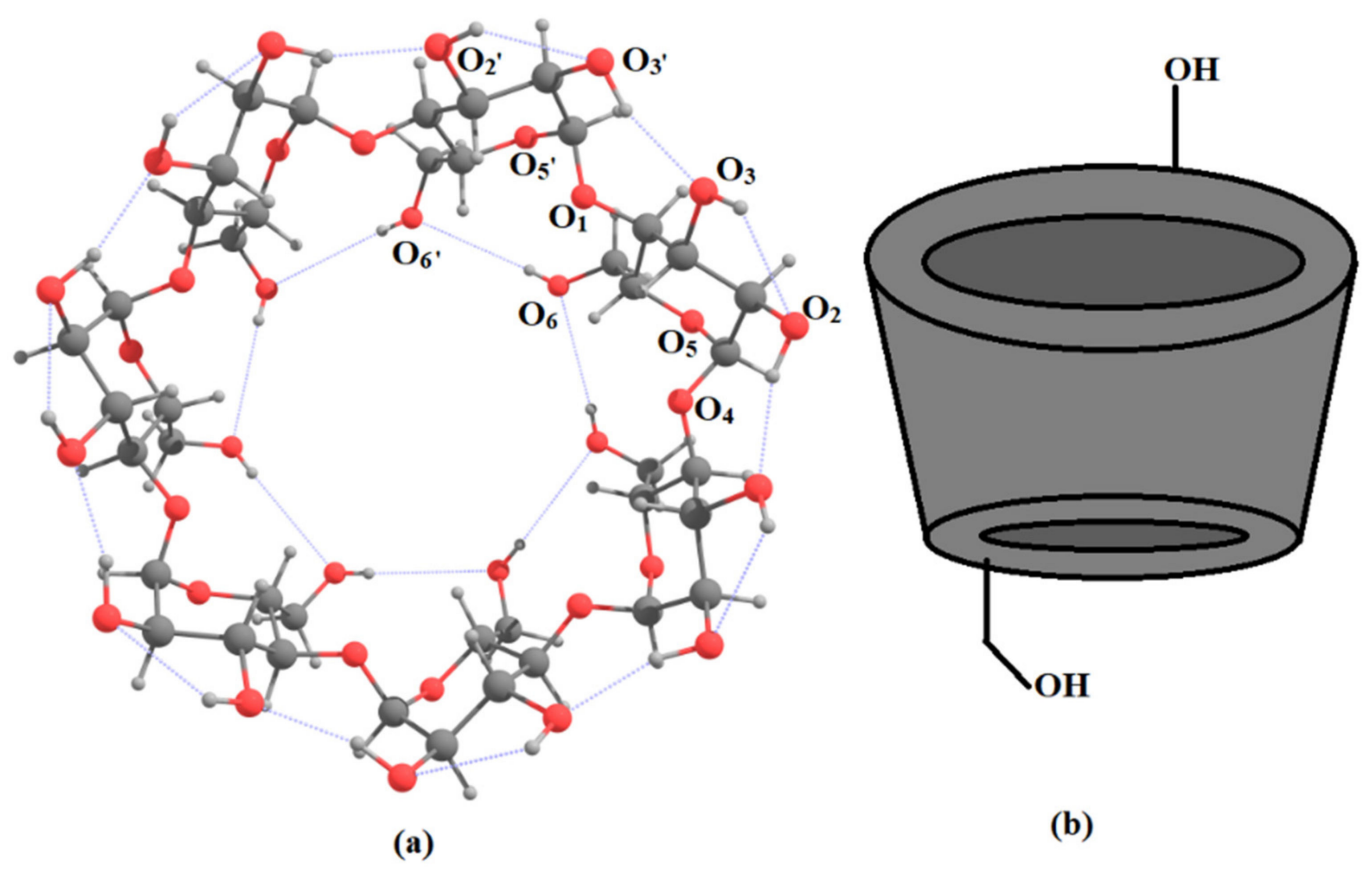
(**a**) A schematic structure of β-cyclodextrin molecule (containing seven glucose units) indicating different types of hydroxyl groups and (**b**) a CD bowl, secondary hydroxyl groups at smaller and primary ones at the larger rim, respectively. See text for details.

Another molecular system wherein the D-glucose units are joined to each other by 1–4 linkage is cyclodextrins (CDs), which are macrocyclic oligosaccharides. They possess a unique ability to entrap guest molecules in their cavities owing to their bucket/bowl shape. Such inclusion complexes are used in the pharmaceutical industry for a variety of formulations. The most commonly known CDs are α-, β- and γ-CDs which have six, seven, and eight glucose units, respectively (see Figure 3). The IHB’s between the secondary O–H groups of glucose units result in the formation of a smaller rim of the CD bowls, whereas the primary O–H groups form the larger ones. The strength of the IHBs is suggested to be an important factor governing the stability of the CDs [94,95,96]. Moreover, one would expect a larger cooperativity contribution due to the formation of a more extended network of HBs between different types of O–H groups. The estimated IHB energies obtained by using the MTA-based method [86] belonged to a wide range of 1.1 to 8.3 kcal/mol at the B3LYP/6-311++G(d,p) level, suggesting that strong HBs are seen in CDs. For the O_6_H···O_6_^′^ HBs, HB energies fall between 6.7 to 8.3 kcal/mol, and for O_3_H···O_2_^′^ HBs, they are between 3.3 to 5.5 kcal/mol and 1.9 to 2.8 kcal/mol for O_2_H···O_3_ HBs. The cooperativity contribution by the O_6_H···O_6_^′^ HB is larger (1.3 to 2.7 kcal/mol) than that for O_3_H···O_2_ (0.3 to 1.0 kcal/mol) and O_2_H···O_3_ (0.25 to 1.10 kcal/mol) HBs. Note that the cooperativity contribution in glucopyranose (0.1 to 1.1 kcal/mol) is much smaller than that in CDs. The higher HB strength in CDs could be one of the possible reasons for the much lower aqueous solubility of the natural CDs than that of comparable acyclic polysaccharides [97,98,99].

We now discuss the HB energies and cooperativity contributions calculated by using MTA in yet another macrocyclic system, viz., *p*-substituted Calix[n]arenes CX[n] (*n* = 4, 5), for exploring substitution effect on the strength of the HBs [87]. The estimated HB energies were between 4.6 to 8.2 kcal/mol, with cooperativity contribution being 0.2 to 2.6 kcal/mol, both calculated at B3LYP/6-311G(d,p) level of theory. The estimated HB energies showed [87] the following order: CX[n]-t-Bu ≈ CX[n]-NH_2_ > CX[n]-CH_2_Cl > CX[n] > CX[n]-SO_3_H > CX[n]-NO_2_ > S-CX[n]-t-Bu > S-CX[n]. It was also observed that the HB energies in CX[5] derivatives are larger than those in CX[4]. Additionally, as expected, large HB energy values and the respective cooperativity contributions were found in CX[n] hosts with electron-donating substituents.

**Figure 4:**
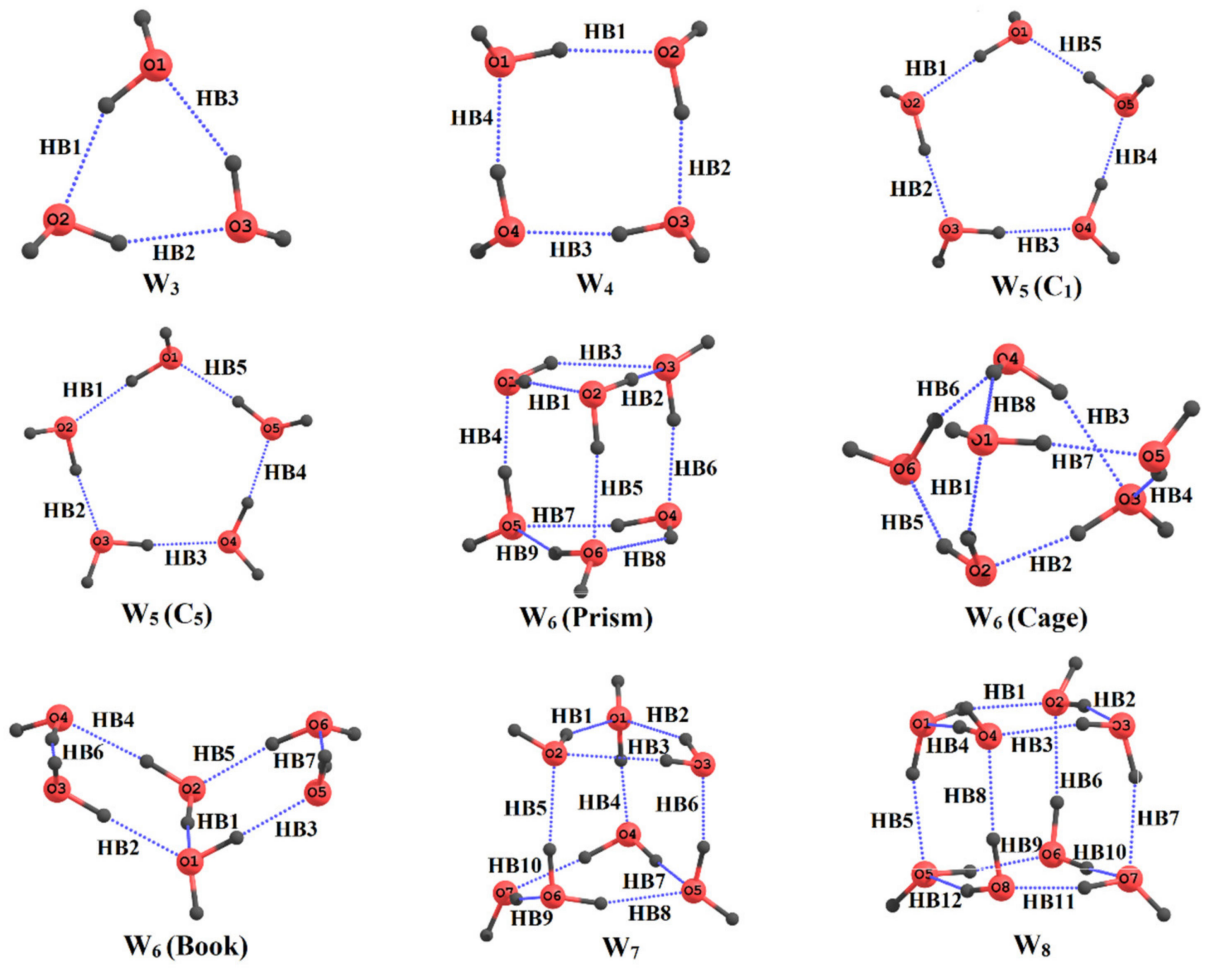
The MP2-optimized geometries of various water clusters (W_n_). Figure reproduced from Ref. [88] with the permission from American Chemical Society (ACS). *Copyright (2020)* *The American Chemical Society*.

Recently, we have widened the application of the MTA-based method for the estimation of individual O–H···O HB energy and their cooperativity contribution in water clusters [88]. Many works reported in the literature focus on the global minimum structure of water clusters of variable sizes [73,75,76,100,101,102,103]. In spite of a large number of studies, the nature of water clusters at the molecular level is not fully understood. For instance, the strengths of individual O-H···O HB interactions reported in the literature are mostly limited to the water–dimer. Larger clusters are expected to have a cooperativity contribution due to the networking of HBs therein. We recently applied the MTA-based method for the reliable estimation of individual O–H···O HB energies and their cooperativity contribution in small water clusters W_n_, *n* = 3 to 8 (see Figure 4).

The fragmentation procedure for the estimation of O–H···O HB energy in W_n_ is similar to the one for molecules, discussed in the previous section. The difference is that no dummy atoms are needed here because no covalent bond is cut. In the present case, two water molecules involved in the formation of an O-H···O HB were removed for generating the two primary fragments keeping the other water molecules within the cluster intact (see Ref. [88]). The calculated HB energies in W_n_, for n = 3 to 8, are in the wide range of 0.3 to 10.7 kcal/mol at the CCSD(T)/aug-cc-pVDZ level, with the respective cooperativity contribution being 1.2 to 7.0 kcal/mol. To check the reliability of the results, the sum of all the HB energies for a given cluster was added to the sum of monomers energies. The molecular energy of a water cluster thus estimated agreed well with the actual energy (typical error less than 8.3 mH), suggesting HB energies obtained by our MTA-based method [88] are reliable.

## 6. Applications to Biomolecules

**Figure 5:**
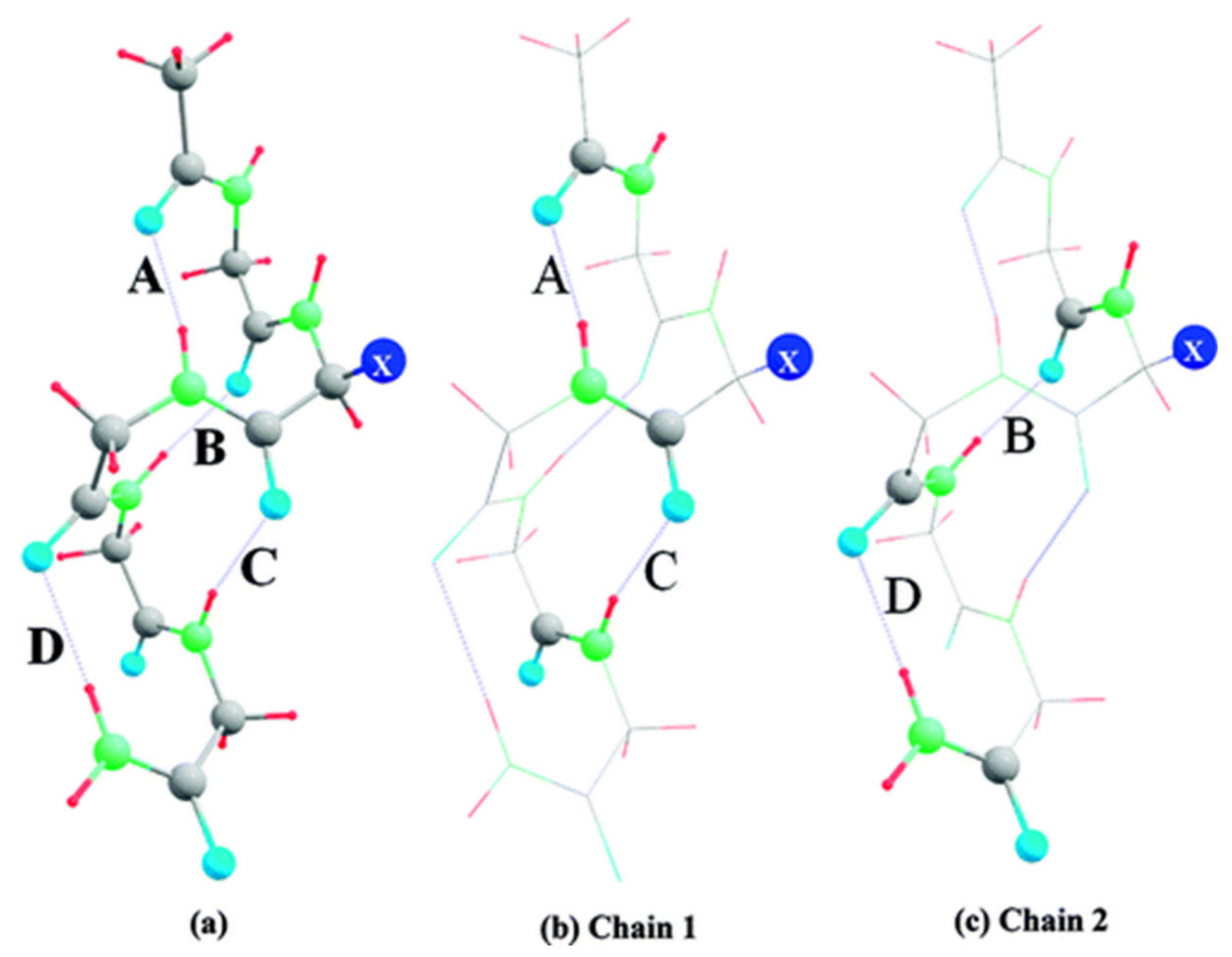
Hydrogen-bonded chains in the helical peptide structures. “X” represents the substituent at the second position. A, B, C and D are four hydrogen bonds. See text for details. Reprinted from Ref. [89] with permission from American Chemical Society. *Copyright (2009) The American Chemical Society.*

We now discuss the application of the MTA-based method for the energy estimation of the N–H···O=C, N–H···N, N–H···S, N–H···O, C–H···N, and resonance-assisted O–H···O=C HBs in biomolecules. Quantitative estimates of the strengths of such individual HB interactions are scarcely available, although they play a vital role in the determination of the structure of biomolecules such as polypeptides/proteins. In order to gauge the strengths of the N–H···O=C HBs, a model tetrapeptide was taken as a test case [89]. It has two different N–H···O=C HBs, the fragmentation scheme being similar to that discussed earlier. In the present case, we remove a –(H)N–(O=C)- functional group of amino acids, involved in the formation of N–H···O=C HB to generate the primary fragments (see Ref. [89]). The MTA-based methodology is applied for the estimation of these IHB energies in five different substituted tetrapeptides of polyglycine abbreviated as GGGGG apart from the capped acetyl and NH_2_ groups. The five substituted tetrapeptides (viz., GAGGG, GVGGG, GLGGG, and GIGGG) in which the second amino acid residue is replaced by alanine (A), valine (V), leucine (L), and isoleucine (I), respectively, were considered (see Figure 5). The corresponding completely substituted tetrapeptides, AAAAA, VVVVV, LLLLL, and IIIII, were also employed with a view to addressing the effect of substituents on the strengths of the different types of N–H···O=C HBs (Chain 1 and Chain 2 in Figure 5). The estimated HB energies at B3LYP/D95(d,p) level were in the range of 4 to 6 kcal/mol in the partially and fully substituted polypeptides. These values were in concurrence with the geometric parameters and reflected the subtle substituents effects for the substituted polypeptides. The MTA-based procedure was thus considered to be applicable for the IHB energy estimation in polypeptides and it would be fascinating to apply it to an actual protein.

**Figure 6:**
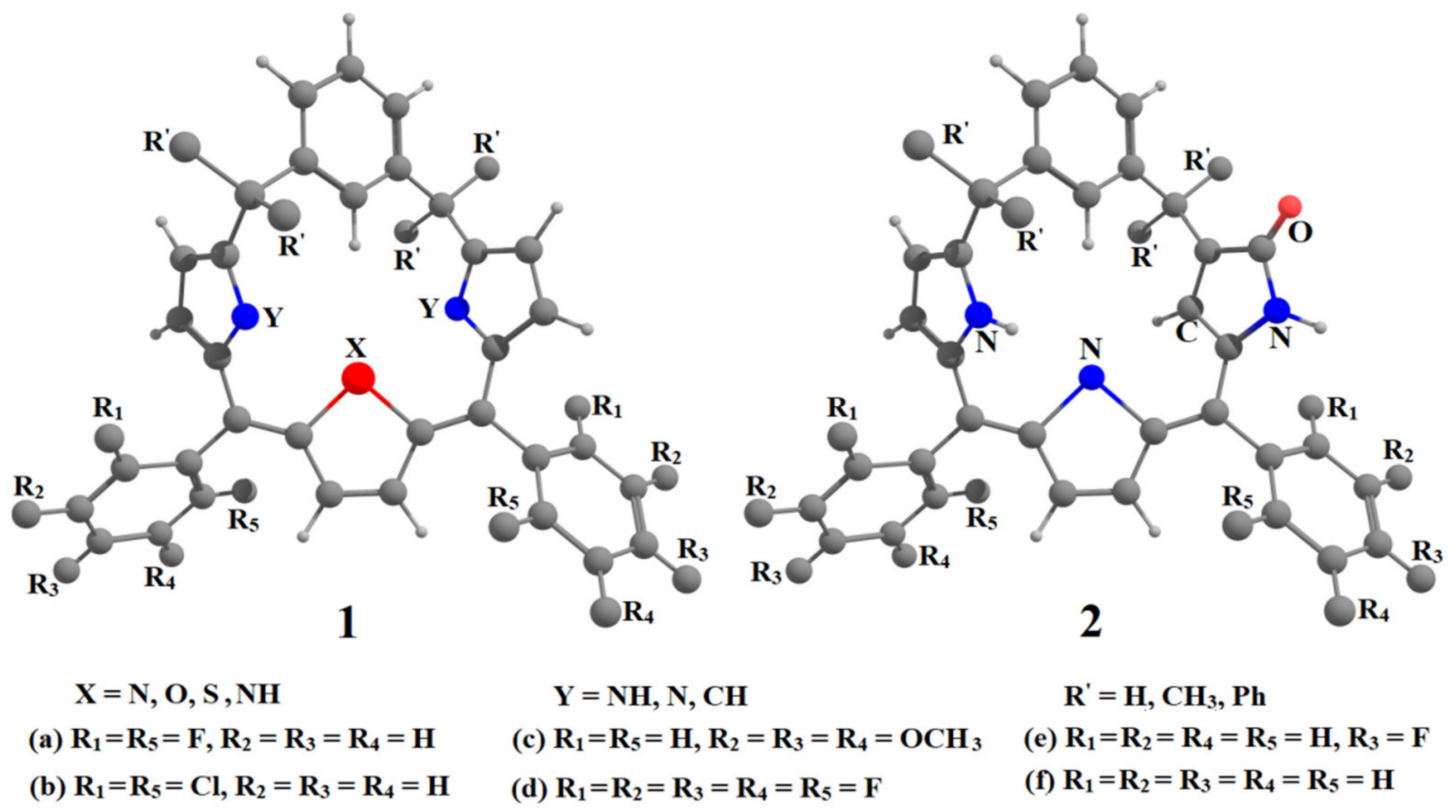
Structures of meta-benziporphodimethene **1** and N-confused meta-benziporphodimethene containing γ-lactam ring isomer (O-up, **2**). See text for details.

Recently, the MTA-based procedure was applied to another biologically important class of molecules, viz., porphyrin analogs called meta-benziporphodimethenes (**1**) and N-confused isomers containing γ-lactam ring (**2** and **3**) [90,91]. In γ-lactam-containing isomers **3** (not shown in Figure 6) the ring O-atom of lactam ring is down, whereas, in isomer **2**, O-atom is up (see Figure 6). The substitution at meso *sp^3^* (R′) and on the benzene at *sp^2^* carbons (R_1_ to R_5_) may affect the strengths of N–H···N, N–H···S, N-H···O, C-H···N HBs, which, in turn, decide the stability of these conformers. Hence, the IHB strengths in substituted **1**, **2**, and **3** were determined by the MTA-based methodology. The estimated N–H···N HB energies were between 11.0 to 15.6 kcal/mol at B3LYP/6-311+G(d,p) level, the individual bifurcated N–H···N interactions being ~6.0 kcal/mol. The N-H···O (~10.0 kcal/mol) and N–H···S (11.2 kcal/mol) HBs were found to be weaker than the N–H···N HBs, with the C-H···N HBs being the weakest (0.1 to 4.4 kcal/mol) [90]. The substituent effect on IHB strength was also investigated [90,91].

It has been suggested that intramolecular O–H···O and resonance-assisted (RA) O–H···O=C HBs may have a pronounced effect on the antioxidant activity of the natural products [104,105,106,107]. Hence, an attempt was made to obtain reliable estimates of IHB energies in these molecules [92]. The presence of an RA IHB was seen in all the antioxidant molecules studied. In some molecules, this type of HB was found to be in the nature of two bifurcated HBs along with IHBs such as O–H···OCH_3_, O–H···OH, and O–H···O(ring) also being present. The energies of O–H···OCH_3_ HBs were found to lie between 2.2 to 2.4 kcal/mol, with the O–H···O (ring) HB energies being much smaller (~0.5 to 0.6 kcal/mol) at the MP2/6-311G(d,p) level. The energy of RA O–H···O=C IHB being largest (10.0 to 17.9 kcal/mol.) Recently, Restropo et al. [108] suggested that the weak HBs are directly related to the antioxidant properties of these molecules. The reliable estimates of the strengths of these weak HBs by the MTA-based method could be useful for quantitative structure–activity relationship (QSAR) studies on a larger set of antioxidant molecules.

## 7. Use of the MTA-Based Method by Other Researchers

We summarize here the use of our MTA-based method for the estimation of IHB energy by other researchers [109,110,111,112,113,114,115,116,117]. In an early use of the method, Lopes Jesus et al. [109] studied the 65 local minima conformers of 1,4-butanediol molecule. The H···O IHB energies in the two lowest energy conformers (c1, c2) were estimated using the MTA method to be 5.5 and 4.1 kcal/mol, respectively. The IHB energies were also obtained by two other methods, viz., conformational analysis (CA) [33,41,42] and use of the empirical Iogansen equation (IE) to spectroscopic data [110]. The IHB energy values turned out to be CA: (4.5 and 3.1 kcal/mol) and IE: (4.1 and 2.2 kcal/mol), in good qualitative agreement with their MTA counterparts.

Rusinska-Roszak et al. extensively used the MTA-based method [111,112,113]. In an early work [111], they estimated the O–H···O=C IHB energies in several aliphatic systems. However, their fragmentation scheme consisted of two appropriately made primary fragments and an overlapping fragment. The IHB energy was estimated, by using the energies of these three fragments and the actual energy of the parent system, ignoring the ternary and quaternary overlap fragments. The estimated IHB energies at MP2/6-311++(2d,2p) level in the saturated hydroxyl compounds were between 1.4 to 7.0 kcal/mol and 13.7 kcal/mol in the unsaturated ones [111].

In another work [112], they estimated the O−H···O=C IHB energies in 299 structures of hydroxycarbonyl aliphatic compounds involving resonance-assisted HB. The estimated IHB energies showed a wide range, from 8.2 to 26.3 kcal/mol. The HB energies showed a good qualitative correlation with other indirect measures of HB strength, viz., geometrical parameters, the O–H stretching frequencies, and the MED values at the BCP [112].

These authors [113] also applied the MTA-based method for the estimation of Ar–O–H···O=C HBs in mono-, di-, and triphenols substituted (by electron-donating/withdrawing groups) at the ortho- position by carbonyl-containing functional groups. The estimated HB energies for phenolic O–H···O=C (six-membered ring) fall in the range of 5.4 to 15.4 kcal/mol at MP2/6-311++G(2d,2p) level. These HBs energies are smaller (4.6 to 9.6 kcal/mol) when the O=C group is a part of seven or eight-membered rings. These HBs’ energy range is similar to corresponding HBs involving saturated carbonyl substituted alcohols.

Very recently, Afonin et al. [114] applied our method for estimating the energy of push–pull effect in *β*-diketones. In this push–pull system, the intramolecular charge transfer (ICT) occurs as a result of interaction between the π-donor and -acceptor parts, joined by a π linker. Further, the IHB is also present in the *Z*-conformation of *β*-diketones. Here, basic idea is to estimate the π component of conjugation energy in these systems. For this purpose, an *E* conformation of the parent *β*-diketones was considered wherein this HB is not present. The molecule in this configuration was fragmented using the MTA-based method. The donor and acceptor groups involved in ICT interaction were separated into two primary fragments, F1 and F2, and the overlapping fragment, F3, is the conjugation unit connecting these groups. The energy expression for the π conjugation energy is similar to that discussed in Section 3, viz., E*_π_*(MTA) = [(E_F1_ + E_F2_ − E_F3_) − E_M_], E_M_ being the energy of the parent molecule in *E* configuration. The effect of electron-donating and -withdrawing groups on the π component of conjugation energy was also estimated. The authors stated [114] that “although the choice of the fragmentation scheme and the computational protocol used did not play a decisive role in estimating the energies of the same IMHB, this issue also needs to be investigated when estimating the conjugation energy.”

Afonin et al. [115] applied the methodology for the quantitative decomposition of RAHB energy in β-diketones into resonance and hydrogen bonding components. They also compared the estimated HB energy by the MTA method with that obtained by the functional-based approach (FBA). In FBA, the HB energy is written as an empirical function of HB descriptors, E_HB_ = f(D), the parameter D is one of the H-bond descriptors (i.e., geometrical, topological, and/or spectral characteristics of the H-bond). For details of these empirical FBA equations, see Ref. [115]. It has been shown by these authors that the FBA method evaluates “the component of RAHB interaction corresponding to the energy of the pure H-bond without resonance component.” However, the MTA method implicitly takes into account the π component in the RAHB interaction and hence “the difference in the energy of the IMHB as evaluated by means of the MTA and FBA yields a quantitative estimate of the resonance component in the case of the resonance-assisted hydrogen bonds” [115]. The resonance component energy was reported to be 6 to 7 kcal/mol for the weak to strong RAHB, respectively. The HB component varied in the wide range from 2 to 20 kcal/mol in a series of the β-diketone molecules [115]. In summary, the energy of IHB by the MTA-based method provides reliable values for the RAHB, including both the resonance and HB components.

Recently the IHB energies estimated by the MTA-based method were compared with those obtained by other indirect HB descriptors for the wide range of malonaldehyde derivatives by Nowroozi et al. [116]. The latter included structural, spectroscopic, topological, and molecular orbital parameters in the intramolecular RAHBs. The substituent effect of electron-donating and -accepting groups on the HB energy was also estimated by the MTA-based method [116]. Further, the significance of π-electron delocalization (π-ED) of RAHB rings was evaluated by the geometrical factor and the harmonic oscillator model of aromaticity (HOMA). The authors [116] stated that “the excellent linear correlations with MTA energies, which may be implied on the validity of RAHB theory”. Further, it was emphasized by authors that “On the basis of these results, one can claim that the MTA method is reliable for estimation of IMHB energies of RAHB systems.”

A significant application of our MTA-method was in the determination of the push–pull π^+^/π^-^ (PPππ) effect in the Henry reaction [117], an organocatalytic reaction catalyzed by squaramide [118]. In this reaction, several noncovalent interactions such as HBs, π···H, π···O, etc. were observed between the squaramide and benzaldehyde. It was suggested that the reaction proceeds via an unprecedented mode of activation, modulated by π···H (π···δ^+^) and π···O (π···δ^-^) interactions formed with the two rings of a naphthyl group and benzaldehyde. These authors employed the MTA-based approach for determining the energies of these two π interactions [118]. Here, the naphthyl group involved in the PPππ interactions, observed in the intermediates (INTs) and transition state (TS) structures, along the most favorable pathway (P1), was replaced with an H atom. Thus, generated INTs and TS structures along the modeled pathway (P1–H) have the same noncovalent interactions as P1, except the two π interactions whose energy is to be determined. The interaction energy of these two π interactions in the INTs and TS structures were estimated as the energy difference in the total interaction energies between the catalyst and the substrates in P1 and that in modeled P1−H pathways. The estimated sum of two π interactions in INTs and TS were found to be between 2.7 to 4.7 kcal/mol at the ωB97X-D level of theory. In summary, the MTA-based method proposed by us has been successfully employed by several other active research groups for exploring the strengths of IHBs and other intramolecular noncovalent interactions in a variety of systems.

## 8. Summary and Concluding Remarks

This review article has summarized a direct and simple procedure for the estimation of X–H···Y IHB energy employing a fragmentation method, viz., the molecular tailoring approach (MTA). It has been applied to a variety of systems having multiple HBs over the last one and half decades. A plus point of the method is that it provides the reliable energy of every individual HB and can also estimate the corresponding cooperativity contribution as a result of interconnected networks of HBs. In this present review article, we have discussed the application of the MTA-based method for the estimation of X–H···Y (X–H = O–H, N–H, C–H, etc. and Y = N, O, S, OH, OCH_3_, O=C, etc.) HB energies in a variety of systems. The systems covered ranged from small alkanediols to large systems such as cyclodextrins and biomolecules such as polypeptides, meta-benzoporphodimethenes, and antioxidant molecules. Further, being of general nature, our method has been utilized as a standard method for a reliable estimation of HB energies by other research groups. It may be emphasized here that the MTA-based method is applicable for the estimation of other noncovalent interactions as well. In recent years, this approach has been applied for the estimation of O–H···O HB energies in water clusters, the π component of the conjugation energy in resonance-assisted, hydrogen-bonded push–pull systems, etc. In a recent impressive application, the method is employed for determining the favorable organocatalytic reaction pathways [118].

It may be noted that the present method can, in principle, be applied to the estimation of IHB energy in larger molecular systems. However, with the increase in the size of a molecule, the size of the fragments would also increase, making the evaluation of HB energies computationally rather demanding. This difficulty can, in principle, be overcome by the use of the MTA methodology discussed in Section 2. For instance, the energy of the parent and fragment molecules can be reliably calculated using MTA at a correlated level of theory which may be further used for estimating the HB energies in larger molecular systems.

Being general in nature, the MTA-based method can be employed for exploring other intramolecular interactions such as π···π [119], C-H···π [120], the so-called halogen bonds [121], dihydrogen bonds [122], sulfur bonds [123], metal-H···S and metal-H···Se bonds [124], etc. Although identified in the recent literature by these labels, they are cut from the same cloth called the *non-covalent interactions*. The prowess of the MTA-based method is that it can be applied to all such inter- and intramolecular interactions.

## Figures and Tables

**Table 1:** The H-bond (HB) distances (in Å), HB energies (in kcal/mol), and the error in the molecular energy estimation for alkanetriols using similar fragments, ΔE=|E_M_ − E_e_|. The corresponding O–H stretching frequencies (cm^−1^) and the molecular electron density (MED) value at the (3, −1) bond critical point (BCP) (a.u.) are also shown. The calculations are performed at MP2 (full)/6-311++G(2d,2p) level theory.

Molecule	HB Label	HB Distances ^a^ (in Å)	HB Energy (kcal/mol)	ΔE (kcal/mol)	O–H Stretch Frequency (cm^−1^)	MED at (3, −1) BCP (a.u.)
1,2,3-propanetriol ^b^	HB1 HB2 HB3	2.16 2.08 2.58	1.90 2.47 1.63	0.50	3784 3765 3845	0.0201
1,2,3-butanetriol ^b^	HB1 HB2 HB3	2.13 2.05 2.58	2.13 2.72 1.60	0.50	3768 3745 3844	0.0211
1,2,4-butanetriol	HB1 HB2	1.98 2.22	2.90 1.75	0.40	3789 3828 3875	0.0219
1,2,5-pentanetriol	HB1 HB2	1.80 2.25	4.97 1.78	0.55	3669 3825 3865	0.0334
1,3,5-pentanetriol	HB1 HB2	1.94 1.96	2.91 2.90	0.58	3763 3792 3875	0.0225 0.0239
2,3,4-pentanetriol ^b^	HB1 HB2 HB3	2.12 2.02 2.56	2.18 2.94 1.50	0.52	3759 3731 3820	0.0223
2,4,6-heptanetriol	HB1 HB2	1.92 1.93	3.02 2.94	0.65	3753 3773 3857	0.0250 0.0242

^a^ The MP2 (FC)/6-311++G(2d,2p) optimized geometries were employed. Table 1 is partially reproduced from our earlier study reported in Ref. [66]; *Copyright (2006) The American Chemical Society.* ^b^ The triols wherein three OH groups are present on the successive C-atoms show three H-bonds. See text for details.

## Data Availability

The data presented in this study are available on request from the corresponding authors.

## References

[1] Pauling L. (1939). The Nature of the Chemical Bond.

[2] Jeffery G.A. (1997). An Introduction to Hydrogen Bonding.

[3] Desiraju G.R., Steiner T. (1999). The Weak Hydrogen Bond. Structural Chemistry and Biology.

[4] Scheiner S. (1997). Hydrogen Bond: A Theoretical Perspective.

[5] Huggins M.L. (1936). Hydrogen Bridges in Ice and Liquid Water. J. Phys. Chem..

[6] Huggins M.L. (1936). Hydrogen Bridges in Organic Compounds. J. Org. Chem..

[7] Nernst W. (1892). Über die Löslichkeit von Mischkrystallen. Z. Phys. Chem..

[8] Werner A. (1902). Ueber Haupt und Nebenvalenzen und die Constitution der Ammoniumverbindungen. Liebigs Ann. Chem..

[9] Moore T.S., Winmill T.F. (1912). The State of Amines in Aqueous Solution. J. Chem. Soc..

[10] Latimer W.M., Rodebush W.H. (1920). Polarity and Ionization from the Standpoint of the Lewis Theory of Valence. J. Am. Chem. Soc..

[11] Barnes W.H. (1929). The Crystal Structure of Ice between 0 °C and −183 °C. Proc. R. Soc. Lond. A.

[12] Huggins M.L. (1971). 50 Years of Hydrogen Bond Theory. Angew. Chem. Int. Ed. Engl..

[13] Pimentel G.C., McClellan A.L. (1960). The Hydrogen Bond.

[14] Arunan E., Desiraju G.R., Klein R.A., Sadlej J., Scheiner S., Alkorta I., Clary D.C., Crabtree R.H., Dannenberg J.J., Hobza P. (2011). Defining the Hydrogen Bond: An Account (IUPAC Technical Report). Pure Appl. Chem..

[15] Pauling L. (1931). The Nature of the Chemical Bond. Applications of Results Obtained from the Quantum Mechanics and from a Theory of Paramagnetic Susceptibility of the Structure of Molecules. J. Am. Chem. Soc..

[16] Chaplin M.F., Lynden-Bell R.M., Morris S.M., Barrow J.D., Finney J.L., Harper C. (2010). Water and Life: The Unique Properties of H_2_O.

[17] Laage D., Elsaesser T., Hynes J.T. (2017). Water Dynamics in the Hydration Shells of Biomolecules. Chem. Rev..

[18] Bellissent-Funel M.-C., Hassanali A., Havenith M., Henchman R., Pohl P., Sterpone F., van der Spoel D., Xu Y., Garcia A.E. (2016). Water Determines the Structure and Dynamics of Proteins. Chem. Rev..

[19] Keutsch F.N., Cruzan J.D., Saykally R.J. (2003). The Water Trimer. Chem. Rev..

[20] Sánchez-Garcȋa E., George L., Montero L.A., Sander W. (2004). 1:2 Formic Acid/Acetylene Complexes:  Ab Initio and Matrix Isolation Studies of Weakly Interacting Systems. J. Phys. Chem. A.

[21] Munshi P., Row T.N.G. (2005). Exploring the Lower Limit in Hydrogen Bonds: Analysis of Weak C−H···O and C−H···π Interactions in Substituted Coumarins from Charge Density Analysis. J. Phys. Chem. A.

[22] Cockroft S.L., Hunter C.A., Lawso K.R., Perkins J., Urch C.J. (2005). Electrostatic Control of Aromatic Stacking Interactions. J. Am. Chem. Soc..

[23] Grabowski S.J. (2004). Hydrogen Bonding Strength-Measures Based on Geometric and Topological Parameters. J. Phys. Org. Chem..

[24] Bellamy (1968). Advances in Infrared Group Frequencies.

[25] Glasel J.A., Frank F. (1982). Water: A Comprehensive Treatise.

[26] Hobza P., Havlas Z. (2000). Blue-Shifting Hydrogen Bonds. Chem. Rev..

[27] Fuster F., Silvi B. (2000). Does the Topological Approach Characterize the Hydrogen Bond?. Theor. Chem. Acc..

[28] Espinosa E., Alkorta I., Elguero J., Molins E. (2002). From Weak to Strong Interactions: A Comprehensive Analysis of the Topological and Energetic Properties of the Electron Density Distribution Involving X–H⋯F–Y Systems. J. Chem. Phys..

[29] Klein R.A. (2003). Hydrogen Bonding in Diols and Binary Diol–Water Systems Investigated using DFT Methods. II. Calculated Infrared OH-Stretch Frequencies, Force Constants, and NMR Chemical Shifts Correlate with Hydrogen Bond Geometry and Electron Density Topology. A Reevaluation of Geometrical Criteria for Hydrogen Bonding. J. Comput. Chem..

[30] Deshmukh M.M., Sastry N.V., Gadre S.R. (2004). Molecular Interpretation of Water Structuring and Destructuring Effects: Hydration of Alkanediols. J. Chem. Phys..

[31] Kovács A., Szabo A., Hargittai I. (2002). Structural Characteristics of Intramolecular Hydrogen Bonding in Benzene Derivatives. Acc. Chem. Res..

[32] Chung G., Kwon O., Kwon Y. (1997). Theoretical Study on 1,2-Dihydroxybenzene and 2-Hydroxythiophenol:  Intramolecular Hydrogen Bonding. J. Phys. Chem. A.

[33] Rozas I., Alkorta I., Elguero J. (2001). Intramolecular Hydrogen Bonds in ortho-Substituted Hydroxybenzenes and in 8-Susbtituted 1-Hydroxynaphthalenes:  Can a Methyl Group Be an Acceptor of Hydrogen Bonds?. J. Phys. Chem. A.

[34] Lipkowski P., Koll A., Karpfen A., Wolschann P. (2002). An Approach to Estimate the Energy of the Intramolecular Hydrogen Bond. Chem. Phys. Lett..

[35] Buemi G., Zuccarello F. (2002). Is the Intramolecular Hydrogen Bond Energy Valuable from Internal Rotation Barriers?. J. Mol. Struct. Theochem..

[36] Korth H.-G., de Heer M.I., Mulder P. (2002). A DFT Study on Intramolecular Hydrogen Bonding in 2-Substituted Phenols:  Conformations, Enthalpies, and Correlation with Solute Parameters. J. Phys. Chem. A.

[37] Kjaergaard H.G., Howard D.L., Schofield D.P., Robinson T.W., Ishiuchi S., Fujii M. (2002). OH- and CH-Stretching Overtone Spectra of Catechol. J. Phys. Chem. A.

[38] Zhang H.-Y., Sun Y.-M., Wang X.-L. (2003). Substituent Effects on O-H Bond Dissociation Enthalpies and Ionization Potentials of Catechols: A DFT Study and Its Implications in the Rational Design of Phenolic Antioxidants and Elucidation of Structure–Activity Relationships for Flavonoid Antioxidants. Chem. Eur. J..

[39] Bakalbassis E.G., Lithoxoidou A.T., Vafiadis A.P. (2003). Theoretical Calculation of Accurate Absolute and Relative Gas- and Liquid-Phase O-H Bond Dissociation Enthalpies of 2-Mono- and 2,6-Disubstituted Phenols, Using DFT/B3LYP. J. Phys. Chem. A.

[40] Jabloński M.A. (2020). Critical Overview of Current Theoretical Methods of Estimating the Energy of Intramolecular Interactions. Molecules.

[41] Jabloński M., Kaczmarek A., Sadlej A.J. (2006). Estimates of the Energy of Intramolecular Hydrogen Bonds. J. Phys. Chem. A.

[42] Jabloński M. (2010). Full vs. Constrained Geometry Optimization in the Open-Closed Method in Estimating the Energy of Intramolecular Charge-Inverted Hydrogen Bonds. Chem. Phys..

[43] Estacio S.G., Cabral do Counta P., Costa Cabral B.J., Minas Da Piedade M.E., Martinho Simoes J.A. (2004). Energetics of Intramolecular Hydrogen Bonding in Di-substituted Benzenes by the ortho-para Method. J. Phys. Chem. A.

[44] Hammett L.P. (1937). The Effect of Structure upon the Reactions of Organic Compounds. Benzene Derivatives. J. Am. Chem. Soc..

[45] Hammett L.P. (1940). Physical Organic Chemistry.

[46] Suresh C.H., Gadre S.R. (1998). A Novel Electrostatic Approach to Substituent Constants: Doubly Substituted Benzenes. J. Am. Chem. Soc..

[47] Hehre W.J., Ditchfield R., Radom L., Pople J.A. (1970). Molecular Orbital Theory of the Electronic Structure of Organic Compounds. V. Molecular Theory of Bond Separation. J. Am. Chem. Soc..

[48] Suresh C.H., Koga N. (2006). An Isodesmic Reaction Based Approach to Aromaticity of a Large Spectrum of Molecules. Chem. Phys. Lett..

[49] Wheeler S.E., Houk K.N., Schleyer P., Allen W.D. (2009). A Hierarchy of Homodesmotic Reactions for Thermochemistry. J. Am. Chem. Soc..

[50] George P., Trachtman M., Brett A.M., Bock C.W. (1977). Comparison of Various lsodesmic and Homodesmotic Reaction Heats with Values derived from Published *Ab initio* Molecular Orbital Calculations. J. Chem. Soc. Perkin Trans..

[51] Howard S.T. (2000). Relationship between Basicity, Strain, and Intramolecular Hydrogen-Bond Energy in Proton Sponges. J. Am. Chem. Soc..

[52] Deshmukh M.M., Suresh C.H., Gadre S.R. (2007). Intramolecular Hydrogen Bond Energy in Polyhydroxy Systems: A Critical Comparison of Molecular Tailoring and Isodesmic Approaches. J. Phys. Chem. A.

[53] Bader R.F.W. (1990). Atoms in Molecules: A Quantum Theory.

[54] Bader R.F.W. (1998). A Bond Path: A Universal Indicator of Bonded Interactions. J. Phys. Chem. A.

[55] Haaland A., Shorokhov D.J., Tverdova N.V. (2004). Topological Analysis of Electron Densities: Is the Presence of an Atomic Interaction Line in an Equilibrium Geometry a Sufficient Condition for the Existence of a Chemical Bond?. Chem. Eur. J..

[56] Poater J., Solà M., Bickelhaupt F.M. (2006). A Model of the Chemical Bond Must Be Rooted in Quantum Mechanics, Provide Insight, and Possess Predictive Power. Chem. Eur. J..

[57] Dem’yanov P., Polestshuk P. (2012). A Bond Path and an Attractive Ehrenfest Force Do Not Necessarily Indicate Bonding Interactions: Case Study on M_2_X_2_ (M = Li, Na, K; X = H, OH, F, Cl). Chem. Eur. J..

[58] Jabłoński M. (2018). Bond Paths Between Distant Atoms Do Not Necessarily Indicate Dominant Interactions. J. Comput. Chem..

[59] Espinosa E., Molins E., Lecomte C. (1998). Hydrogen Bond Strength Revealed by Topological Analyses of Experimentally Observed Electron Densities. Chem. Phys. Lett..

[60] Guevara-Vela J.M., Francisco F., Rocha-Rinza T., Pendás A.M. (2020). Interacting Quantum Atoms-Review. Molecules.

[61] Gatti C., May E., Destro R., Cargnoni F. (2002). Fundamental Properties and Nature of CH···O Interactions in Crystals on the Basis of Experimental and Theoretical Charge Densities. The Case of 3,4-Bis(dimethylamino)-3-cyclobutene-1,2-dione (DMACB) Crystal. J. Phys. Chem. A.

[62] Nikolaienko T.Y., Bulavin L.A., Hovorun D.M. (2012). Bridging QTAIM with Vibrational Spectroscopy: The Energy of Intramolecular Hydrogen Bonds in DNA-related Biomolecules. Phys. Chem. Chem. Phys..

[63] Klein R.A. (2002). Electron Density Topological Analysis of Hydrogen Bonding in Glucopyranose and Hydrated Glucopyranose. J. Am. Chem. Soc..

[64] Klein R.A. (2002). Ab initio conformational studies on diols and binary diol-water systems using DFT methods. Intramolecular hydrogen bonding and 1:1 complex formation with water. J. Comput. Chem..

[65] Howard D.L., Jørgensen P., Kjaergaard H.G. (2005). Weak Intramolecular Interactions in Ethylene Glycol Identified by Vapor Phase OH-Stretching Overtone Spectroscopy. J. Am. Chem. Soc..

[66] Deshmukh M.M., Gadre S.R., Bartolotti L.J. (2006). Estimation of Intramolecular Hydrogen Bond Energy via Molecular Tailoring Approach. J. Phys. Chem. A.

[67] Deshmukh M.M., Bartolotti L.J., Gadre S.R. (2008). Intramolecular Hydrogen Bonding and Cooperative Interactions in Carbohydrates via the Molecular Tailoring Approach. J. Phys. Chem. A.

[68] Gadre S.R., Shirsat R.N., Limaye A.C. (1994). Molecular Tailoring Approach for Simulation of Electrostatic Properties. J. Phys. Chem..

[69] Ganesh V., Dongare R.K., Balanarayan P., Gadre S.R. (2006). Molecular Tailoring Approach for Geometry Optimization of Large Molecules: Energy Evaluation and Parallelization Strategies. J. Chem. Phys..

[70] Elango M., Subramanian V., Rahalkar A.P., Gadre S.R., Sathyamurthy N. (2008). Structure, Energetics, and Reactivity of Boric Acid Nanotubes: A Molecular Tailoring Approach. J. Phys. Chem. A.

[71] Gadre S.R., Ganesh V. (2006). Molecular Tailoring Approach: Towards PC-based ab Initio Treatment of Large Molecules. J. Theor. Comput. Chem..

[72] Rahalkar A.P., Ganesh V., Gadre S.R. (2008). Enabling Ab-initio Hessian and Frequency Calculations of Large Molecules. J. Chem. Phys..

[73] Sahu N., Gadre S.R. (2015). Accurate Vibrational Spectra via Molecular Tailoring Approach: A Case Study of Water Clusters at MP2 Level. J. Chem. Phys..

[74] Sahu N., Gadre S.R. (2016). Vibrational Infrared and Raman Spectra of Polypeptides: Fragments-in-fragments Within Molecular Tailoring Approach. J. Chem. Phys..

[75] Furtado J.P., Rahalkar A.P., Shankar S., Bandyopadhyay P., Gadre S.R. (2012). Facilitating Minima Search for Large Water Clusters at the MP2 Level via Molecular Tailoring. J. Phys. Chem. Lett..

[76] Sahu N., Singh G., Nandi A., Gadre S.R. (2016). Toward an Accurate and Inexpensive Estimation of CCSD (T)/CBS Binding Energies of Large Water Clusters. J. Phys. Chem. A.

[77] Gadre S.R., Yeole S.D., Sahu N. (2014). Quantum Chemical Investigations on Molecular Clusters. Chem. Rev..

[78] Singh G., Nandi A., Gadre S.R. (2016). Breaking the Bottleneck: Use of Molecular Tailoring Approach for the Estimation of Binding Energies at MP2/CBS Limit for Large Water Clusters. J. Chem. Phys..

[79] Yeole S.D., Gadre S.R. (2010). On the Applicability of Fragmentation Methods to Conjugated π-Systems within Density Functional Framework. J. Chem. Phys..

[80] Yeole S.D., Gadre S.R. (2011). Molecular Cluster Building Algorithm: Electrostatic Guidelines and Molecular Tailoring Approach. J. Chem. Phys..

[81] Khire S.S., Gadre S.R. (2019). Pragmatic Many-body Approach for Economic MP2 Energy Estimation of Molecular Clusters. J. Phys. Chem. A.

[82] Khire S.S., Bartolotti L.J., Gadre S.R. (2018). Harmonizing Accuracy and Efficiency: A Pragmatic Approach to Fragmentation of Large Molecules. J. Chem. Phys..

[83] Frisch M.J., Trucks G.W., Schlegel H.B., Scuseria G.E., Robb M.A., Cheeseman J.R., Scalmani G., Barone V., Petersson G.A., Nakatsuji H. (2016). Gaussian 16, Revision A.03.

[84] Siddiqui N., Singh V., Deshmukh M.M., Gurunath R. (2015). Structures, Stability and Hydrogen Bonding in Inositol Conformers. Phys. Chem. Chem. Phys..

[85] Deshmukh M.M., Gadre S.R., Cocinero E.M. (2015). Stability of Conformationally Locked Free Fructose: Theoretical and Computational Insights. New J. Chem..

[86] Deshmukh M.M., Bartolotti L.J., Gadre S.R. (2011). Intramolecular Hydrogen Bond Energy and Cooperative Interactions in α-, β-, and γ-cyclodextrin Conformers. J. Comput. Chem..

[87] Khedkar J.K., Deshmukh M.M., Gejji S.P., Gadre S.R. (2012). Intramolecular Hydrogen Bonding and Cooperative Interactions in Calix[n]arenes (n = 4,5). J. Phys. Chem. A.

[88] Ahirwar M.B., Gadre S.R., Deshmukh M.M. (2020). Direct and Reliable Method for Estimating the Hydrogen Bond Energies and Cooperativity in Water Clusters Wn, n = 3 to 8. J. Phys. Chem. A.

[89] Deshmukh M.M., Gadre S.R. (2009). Estimation of N-H···O=C Intramolecular Hydrogen Bond Energy in Polypeptides. J. Phys. Chem. A.

[90] Kumar A., Hung C.-H., Rana S., Deshmukh M.M. (2019). Study on the Structure, Stability, and Tautomerism of meta-Benziporphodimethenes and N-Confused Isomers Containing γ-Lactam Ring. J. Mol. Struct..

[91] Ahluwalia D., Kumar A., Warkar S.G., Deshmukh M.M. (2020). Effect of Substitutions on the Geometry and Intramolecular Hydrogen Bond Strength in meta-benziporphodimethenes: A New Porphyrin Analogue. J. Mol. Struct..

[92] Singh V., Ibnusaud I., Gadre S.R., Deshmukh M.M. (2020). Fragmentation Method Reveals a Wide Spectrum of Intramolecular Hydrogen Bond Energies in Antioxidant Natural Products. New J. Chem..

[93] Afonin A.V., Vashchenkoa A.V., Sigalov M.V. (2016). Estimating the energy of intramolecular hydrogen bonds from ^1^H NMR and QTAIM calculations. Org. Biomol. Chem..

[94] Lipkowitz K.B. (1998). Applications of Computational Chemistry to the Study of Cyclodextrins. Chem. Rev..

[95] Crini G. (2014). Review: A History of Cyclodextrins. Chem. Rev..

[96] Pinjari R.V., Joshi K.A., Gejji S.P. (2007). Theoretical Studies on Hydrogen Bonding, NMR Chemical Shifts and Electron Density Topography in α, β and γ-Cyclodextrin Conformers. J. Phys. Chem. A.

[97] Higuchi T., Connors K.A., Reilly C.N. (1965). Advances in Analytical Chemistry and Instrumentation.

[98] Loftsson T., Brewster M.E. (1996). Pharmaceutical Applications of Cyclodextrins. 1. Drug Solubilization and Stabilization. J. Pharm. Sci..

[99] Loftsson T., Jarho P., Másson M., Järvine T. (2005). Cyclodextrins in Drug Delivery. Expert Opin. Drug Deliv..

[100] Yoo S., Aprà E., Zeng X.C., Xantheas S.S. (2010). High-Level Ab Initio Electronic Structure Calculations of Water Clusters (H_2_O)_16_ and (H_2_O)_17_: A New Global Minimum for (H_2_O)_16_. J. Phys. Chem. Lett..

[101] Maheshwary S., Patel N., Sathyamurthy N., Kulkarni A.D., Gadre S.R. (2001). Structure and Stability of Water Clusters (H_2_O)_n_, n =8−20: An Ab Initio Investigation. J. Phys. Chem. A.

[102] Kazachenko S., Thakkar A.J. (2009). Improved Minima-Hopping. TIP4P Water Clusters, (H_2_O)_n_ with n ≤ 37. Chem. Phys. Lett..

[103] Sahu N., Gadre S.R., Rakshit A., Bandyopadhyay P., Miliordos E., Xantheas S.S. (2014). Low Energy Isomers of (H_2_O)_25_ from a Hierarchical Method Based on Monte Carlo Temperature Basin Paving and Molecular Tailoring Approaches Benchmarked by MP2 Calculations. J. Chem. Phys..

[104] Foti M.C., Johnson E.R., Vinqvist M.R., Wright J.S., Barclay L.R.C., Ingold K.U. (2002). Naphthalene Diols: A New Class of Antioxidants Intramolecular Hydrogen Bonding in Catechols, Naphthalene Diols, and Their Aryloxyl Radicals. J. Org. Chem..

[105] Foti M.C., Barclay L.R.C., Ingold K.U. (2002). The Role of Hydrogen Bonding on the H-Atom-Donating Abilities of Catechols and Naphthalene Diols and on a Previously Overlooked Aspect of Their Infrared Spectra. J. Am. Chem. Soc..

[106] Amorati R., Lucarini M., Mugnaini V., Pedulli G.F. (2003). Antioxidant Activity of o-Bisphenols: The Role of Intramolecular Hydrogen Bonding. J. Org. Chem..

[107] Martίnez-Cifuentes M., Weiss-López B.E., Santos L.S., Araya-Maturana R. (2014). Intramolecular Hydrogen Bond in Biologically Active o-Carbonyl Hydroquinones. Molecules.

[108] Llano S., Gómez S., Londoño J., Restropo A. (2019). Antioxidant activity of Curcuminoids. Phys. Chem. Chem. Phys..

[109] Lopes Jesus A.J., Rosado M.T.S., Reva I., Fausto R., Eusébio M.E.S., Redinha J.S. (2008). Structure of Isolated 1,4-Butanediol: Combination of MP2 Calculations, NBO Analysis, and Matrix-Isolation Infrared Spectroscopy. J. Phys. Chem. A.

[110] Iogansen A.V. (1999). Direct Proportionality of the Hydrogen Bonding Energy and the Intensification of the Stretching ν(XH) Vibration in Infrared Spectra. Spectrochim. Acta Part A.

[111] Rusinska-Roszak D., Sowinski G. (2014). Estimation of the Intramolecular O−H···O=C Hydrogen Bond Energy via the Molecular Tailoring Approach. Part I: Aliphatic Structures. J. Chem. Inf. Model..

[112] Rusinska-Roszak D. (2015). Intramolecular O−H···O=C Hydrogen Bond Energy via the Molecular Tailoring Approach to RAHB Structures. J. Phys. Chem. A.

[113] Rusinska-Roszak D. (2017). Energy of Intramolecular Hydrogen Bonding in ortho-Hydroxybenzaldehydes, Phenones and Quinones. Transfer of Aromaticity from ipso-Benzene Ring to the Enol System(s). Molecules.

[114] Afonin A.V., Rusinska-Roszak D. (2020). Molecular Tailoring Approach–New Guide to Quantify the Energy of Push-Pull Effect: Case Study on the (E) 3-(1H-pyrrol-2-yl)prop-2-enones. Phys. Chem. Chem. Phys..

[115] Afonin A.V., Vashchenko A.V. (2020). Quantitative Decomposition of Resonance-assisted Hydrogen Bond Energy in β-diketones into Resonance and Hydrogen Bonding (π- and σ-) Components using Molecular Tailoring and Function-based Approaches. J. Comput. Chem..

[116] Keykhaei A., Nowroozi A. (2020). On the Performance of Molecular Tailoring Approach for Estimation of the Intramolecular Hydrogen Bond Energies of RAHB Systems: A Comparative Study. Struct. Chem..

[117] Henry L. (1895). Formation synthétique d’alcools nitrés. Ir. J. Med. Sci..

[118] Alegre-Requena J.V., Marqueés-Loópez E., Herrera R.P. (2017). “Push−Pull π+/π−” (PPππ) Systems in Catalysis. ACS Catal..

[119] Tsuzuki S., Honda K., Uchimaru T., Mikami M., Tanabe K. (2002). Origin of Attraction and Directionality of the π/π Interaction: Model Chemistry Calculations of Benzene Dimer Interaction. J. Am. Chem. Soc..

[120] Mishra B.K., Deshmukh M.M., Ramanathan V. (2014). C-H···π interactions and the nature of the donor carbon atom. J. Org. Chem..

[121] Desiraju G., Ho P.S., Kloo L., Anthony C.L., Marquardt R., Metrangolo P., Politzer P., Resnati G., Rissanen K. (2013). Definition of the halogen bond (IUPAC Recommendations 2013). Pure Appl. Chem..

[122] Grabowski S.J. (2013). Dihydrogen bond and X-H···σ interaction as sub-classes of hydrogen bond. J. Phys. Org. Chem..

[123] Biswal H.S., Wategaonkar S. (2009). Sulfur, not too far behind O, N, and C: SH···π hydrogen bond. J. Phys. Chem. A.

[124] Sahoo D.K., Jena S., Dutta J., Rana A., Biswal H.S. (2019). Nature and strength of M–H···S and M–H···Se (M = Mn, Fe, & Co) hydrogen bond. J. Phys. Chem. A.

